# USF1 and hSET1A Mediated Epigenetic Modifications Regulate Lineage Differentiation and *HoxB4* Transcription

**DOI:** 10.1371/journal.pgen.1003524

**Published:** 2013-06-06

**Authors:** Changwang Deng, Ying Li, Shermi Liang, Kairong Cui, Tal Salz, Hui Yang, Zhanyun Tang, Patrick G. Gallagher, Yi Qiu, Robert Roeder, Keji Zhao, Jörg Bungert, Suming Huang

**Affiliations:** 1 Department of Biochemistry and Molecular Biology, University of Florida College of Medicine, Gainesville, Florida, United States of America; 2 Systems Biology Center, NHLBI, NIH, Bethesda, Maryland, United States of America; 3 Department of Anatomy and Cell Biology, University of Florida College of Medicine, Gainesville, Florida, United States of America; 4 Laboratory of Biochemistry and Molecular Biology, The Rockefeller University, New York, New York, United States of America; 5 Department of Pediatrics, Yale University School of Medicine, New Haven, Connecticut, United States of America; 6 Genetics Institute, University of Florida College of Medicine, Gainesville, Florida, United States of America; Yale University, United States of America

## Abstract

The interplay between polycomb and trithorax complexes has been implicated in embryonic stem cell (ESC) self-renewal and differentiation. It has been shown recently that WRD5 and Dpy-30, specific components of the SET1/MLL protein complexes, play important roles during ESC self-renewal and differentiation of neural lineages. However, not much is known about how and where specific trithorax complexes are targeted to genes involved in self-renewal or lineage-specification. Here, we report that the recruitment of the hSET1A histone H3K4 methyltransferase (HMT) complex by transcription factor USF1 is required for mesoderm specification and lineage differentiation. In undifferentiated ESCs, USF1 maintains hematopoietic stem/progenitor cell (HS/PC) associated bivalent chromatin domains and differentiation potential. Furthermore, USF1 directed recruitment of the hSET1A complex to the *HoxB4* promoter governs the transcriptional activation of *HoxB4* gene and regulates the formation of early hematopoietic cell populations. Disruption of USF or hSET1A function by overexpression of a dominant-negative AUSF1 mutant or by RNA-interference-mediated knockdown, respectively, led to reduced expression of mesoderm markers and inhibition of lineage differentiation. We show that USF1 and hSET1A together regulate H3K4me3 modifications and transcription preinitiation complex assembly at the hematopoietic-associated *HoxB4* gene during differentiation. Finally, ectopic expression of USF1 in ESCs promotes mesoderm differentiation and enforces the endothelial-to-hematopoietic transition by inducing hematopoietic-associated transcription factors, *HoxB4* and *TAL1*. Taken together, our findings reveal that the guided-recruitment of the hSET1A histone methyltransferase complex and its H3K4 methyltransferase activity by transcription regulator USF1 safeguards hematopoietic transcription programs and enhances mesoderm/hematopoietic differentiation.

## Author Summary

Embryonic stem cells (ESCs) are capable of differentiating into any type of cell or tissue of the body. It is important to understand how developmental genes are controlled during differentiation of ESCs into specific cell types. The hSET1/MLL histone modifying protein complexes add methyl groups to lysine 4 on the N-terminal tails of the DNA associated protein histone H3 and activate gene expression. Although the hSET1/MLL enzymatic complexes play a role in activating genes involved in ESC growth and differentiation, how and where these activities are targeted to remains unclear. In this report, we demonstrate that DNA binding factor USF1 interacts with and brings the hSET1A enzymatic complex to its target gene, *HoxB4*, during blood cell specification and differentiation. Consistent with the function of HoxB4 in early blood cell formation, we found that the inactivation of USF1 or hSET1A activities leads to a block in the differentiation of blood cells and causes reductions in methylation levels of H3K4 and expression of *HoxB4*, without impairing the self-renewal capability of ESCs. Taken together, our findings reveal that the collaboration between the hSET1A enzymatic complex and DNA binding regulator USF1 activates developmental genes that control cellular differentiation programs during development.

## Introduction

Embryonic stem cells (ESCs) have the ability to differentiate into any cell type of the body and therefore offer a great tool for studying processes involved in cellular differentiation. ESCs also provide a great potential for application of regenerative medicine that is based on two key properties of stem cells: self-renewal and differentiation. Recent genome-wide chromatin studies revealed that the pluripotency of ESCs is maintained by unique chromatin signatures [1], [2]. To maintain the stemness properties of ESCs, pluripotency associated genes such as *Oct4*, *Sox2*, and *Nanog* are marked by high levels of H3K4me3 whereas many silenced lineage-specific genes are either marked by bivalent H3K4me3/H3K27me3 or by H3K27me3 alone [3]–[9]. In particular, bivalent domains, a unique chromatin feature of stem cells and some differentiated cell lineages, mark developmental genes that are primed to be activated [2]. Bivalent domains were observed in the clusters of *Hox* genes and other genes that are required for early development [3], [10]. Aberrations in *Hox* gene expression often result in abnormal development and malignancy. Although it has been suggested that both polycomb (PcG) and trithorax (TrxG) group complexes play an important role in ESC self-renewal and differentiation [7], [8], [11], [12], the mechanisms by which specific TrxG proteins and the modification of H3K4me3 are targeted to specific gene loci and initiate differentiation of particular cell lineages still remain unknown.

In mammalian cells, the conserved SET domain-containing hSET1/MLL TrxG family complexes specifically methylate histone H3K4 [13]. In addition to the SET domain-containing catalytic subunit, hSET1/MLL complexes comprise several integrated subunits, WDR5, RBBP5, ASH2L, and HCF1, that are required for the enzymatic activity [14], [15]. Deletion of any one of the core subunits drastically reduces global H3K4 methylation [14], suggesting that hSET1/MLL complexes play a critical role in shaping the landscape of global H3K4 methylation. Although they share common structural subunits, the hSET1/MLL complexes contain distinct enzymatic subunits (hSET1A, hSET1B, MLLI, MLL2, MLL3 or MLL4). MLL1 is required for definitive hematopoiesis [16], but loss of *Mll1* reduces H3K4 methylation only at the *HoxC* loci and has little effect on other *Hox* gene loci [17]. In contrast, MLL3/4 has been linked to adipogenesis [18]. These results suggest that the enzymatic subunits of the TrxG complexes may have cell-type specific functions. Furthermore, it has been shown that Dpy-30, a mammalian core subunit of the SET1/MLL-like complex, controls neuronal differentiation of ESCs but not self-renewal [12]. In contrast, WRD5 mediates ESC self-renewal and reprogramming [11]. Both DPY-30 and WDR5 are shared by all of the hSET1/MLL complexes. It is still unknown how integration of different enzymatic subunits of the complex, hSET1A, hSET1B, MLL1, MLL2, MLL3, or MLL4, affects regulation of ESC pluripotency versus lineage differentiation.

During hematopoiesis, *Hox* genes are critical for maintaining the balance between self-renewal and differentiation of hematopoietic stem/progenitor cells (HS/PCs). The *Hox* genes are associated with bivalent domains in undifferentiated ESCs [3]. The sequential expression of *Hox* genes during embryonic development is regulated and maintained epigenetically by PcG and TrxG group regulators [19]. Ectopic induction of *HoxB4* in primitive ESCs leads to hematopoietic cell fate specification [20], [21], suggesting that HoxB4 plays an important role in the switch of the balance between self-renewal and differentiation of ESCs towards the hematopoietic lineage. In addition, HoxB4 has been shown to induce both murine and human hematopoietic progenitor cells and to enhance multilineage hematopoietic engraftment of lethally irradiated mice [22]–[25]. In contrast to the *HoxB4* gene, the anterior *HoxB* genes, *B2*, *B3*, *B5*, and *B6* are dependent on MLL1 for transcriptional activation [26]. How the *HoxB4* gene is dynamically activated to specify ESC fate during early hematopoiesis remains largely unclear. It was reported that USF1 and USF2 heterodimers interact with the *HoxB4* promoter and activate hematopoietic expression of the *HoxB4* gene in response to cytokine-mediated self-renewal and expansion of HSCs [27], [28]. The formation of a NF-Y/USF protein complex is essential for full *HoxB4* promoter activity and a potent inducer of *HoxB4* gene in hematopoietic cells [29], [30]. By protein affinity purification, we found that USF1 is associated with the hSET1A complex and establishes active chromatin boundaries containing high H3K4 methylation levels in erythroid cells [31]. This raises the possibility that although USFs are ubiquitous transcription factors, they might be involved in regulating hematopoietic differentiation by forming active chromatin domains and recruiting transcription complexes at hematopoietic specific genes. The roles of USF1 and hSET1A protein complexes in stem cell fate commitment and differentiation remain to be determined.

Here, we report that the collaboration of USF1 with the hSET1A but not the MLL protein complex is required for mesoderm specification and subsequent hematopoietic cell differentiation. Although hSET1A depletion did not affect ESC self-renewal, it blocks mesoderm differentiation and subsequent hematopoietic differentiation by decreased H3K4me3 levels and transcription preinitiation complex formation at the hematopoietic associated *HoxB4* gene. Transcription factor USF1, which recruits hSET1A, maintains *HoxB4* transcription. Disruption of USF1 prevents hSET1A recruitment, mesoderm development, and further differentiation into hematopoietic cells. Interestingly, Ectopic expression of USF1 enhances mesoderm differentiation and further hematopoietic progenitor formation. Thus, our data reveal that the guided-recruitment of hSET1A and H3K4 methylation patterns by the DNA-binding protein USF1 initiates a lineage transcription program and counteracts ESC self-renewal.

## Results

### ASH2L mediates USF1 and hSET1A complex interaction and *HoxB4* promoter activation

**Figure 1:**
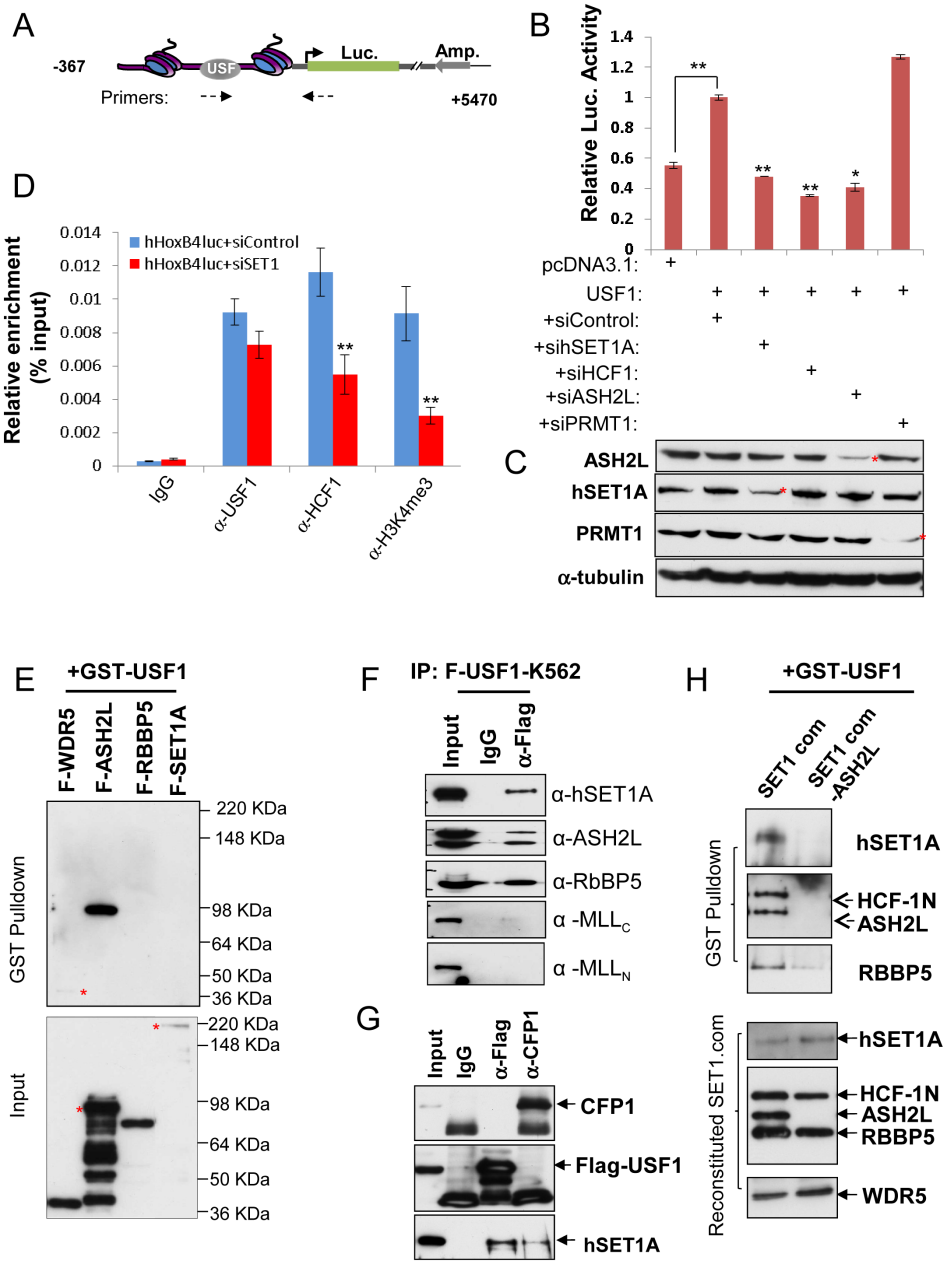
ASH2L mediates USF1 interaction with the hSET1A core complex and *Hox* gene activation. (A) Schematic representation of the *HoxB4* promoter driven pREP4 luciferase episomal reporter construct. (B) K562 cells were transfected with a pREP4-hHoxB4-luc reporter, an expression vector for USF1, and siRNA targeting hSET1A, HCF1, ASH2L or PRMT1. A CMV-driven renilla luciferase plasmid was used as a transfection control. Transfected cells were cultured for 48 hrs and lysed for measurement of luciferase activity. Data are shown as mean ± SD. ** P<0.01; * P<0.05. (C) Western blotting analysis of the levels of ASH2L, hEST1A, PRMT1, and α-tubulin in K562 cells transfected with luciferase reporter and siRNA constructs. (D) ChIP analysis of USF1 binding, HCF1 recruitment, and H3K4me3 association in K562 cells transfected with the pREP4-hHoxB4-luc reporter, an expression vector for USF1, siControl, or siRNA targeting hSET1A. Data are shown as mean ± SD. ** P<0.01. (E)ASH2L of the hSET1A core complex directly interacts with GST-USF1. GST-USF1, pre-absorbed to glutathione-sepharose beads, was incubated with each Flag tagged component of the hSET1A complex and analyzed by WB with Flag antibody (Top). Expression of the hSET1A components purified from virally infected SF9 cells (Bottom). (F)Co-IP assay in K562 cells showing USF1 associates with hSET1A but not with MLL1. (G) Co-IP assay in K562 cells showing that the USF1 associated hSET1A complex does not contain CFP1. (H) Interaction of USF1 with hSET1A in the presence but not absence of ASH2L. GST-USF1 was pre-absorbed to glutathione-sepharose beads and incubated with the purified, baculovirus reconstituted hSET1A complex with or without ASH2L (Top). Immunoblots of the purified, baculovirus expressing hSET1A complex components with or without ASH2L are shown as inputs (Bottom).

We previously showed that affinity purified USF1 complexes contained multiple components of the hSET1A complex and possessed H3K4 histone methyltransferase (HMT) activity to maintain chromatin barrier function [31]. Given that hematopoietic expression of the *HoxB4* gene is also dependent on transcription factor USF1 [27], [30], we sought to examine whether the recruitment of hSET1A complex by USF1 is required for USF1-mediated *HoxB4* promoter activation in hematopoietic cells. A 367 (−276∼+90) bp DNA fragment including the proximal *HoxB4* promoter containing a USF1 binding site was cloned into the episomal pRep4-luciferase vector. This vector was introduced into K562 cells (Figure 1A). Consistent with the positive role of USF1 in *HoxB4* activation, coexpression of USF1 activated the *HoxB4* promoter driven luciferase reporter activity by 1–2 fold compared to the vector control. Expression of dominant negative AUSF1, which interferes with endogenous USF DNA binding, reduced reporter gene activity by 79% compared to the vector control (Figure 1B and S1A–C). Introduction of siRNAs targeting the core components of the hSET1A complex, hSET1A, HCF1, or ASH2L (Figure 1C), resulted in a complete loss of USF1-mediated transcription activation of the *HoxB4* promoter compared to the scrambled siRNA control (Figure 1B). Reduction of PRMT1 expression, which is required for the USF1 mediated activation of a *β-globin* promoter driven reporter in K562 cells (Figure S1C) and expression of the endogenous *β-globin* gene [32], did not affect *HoxB4* promoter activity (Figure 1B). As a control, hSET1A and PRMT1 did not activate HDAC1 promoter driven luciferase reporter activity (Figure S1B) suggesting that hSET1A and PRMT1 may regulate a unique set of target genes. The reduction of reporter activity mediated by hSET1A KD resulted from the loss of hSET1A complex recruitment and H3K4me3 levels at the reporter gene, but not from reduced USF1 binding (Figure 1D). In addition, USF1, the hSET1A complex, and H3K4me3 are associated with and required for *HoxB4* expression in erythroleukemia K562 cells (Figure S1D–F). Taken together, the data suggest that the specific recruitment of the hSET1A complex is critical for USF1 mediated transcriptional activation of the *HoxB4* gene in the hematopoietic lineage.

The hSET1A complex contains several core components. To determine which core subunit, WDR5, ASH2L, RBBP5, or hSET1A, is involved in the direct interaction between USF1 and the hSET1A complex, glutathione-sepharose bead conjugated GST-USF1 was incubated with individual Flag-tagged hSET1A components following baculovirus-mediated expression and purification (Figure 1E Bottom). Although USF1 weakly (if any) associated with WDR5, it strongly interacted with ASH2L (Figure 1E Top). As a control, GST alone did not interact with ASH2L (Figure S1G and S1H). Furthermore, the deletion analysis showed that the basic helix-loop-helix domain of USF1 mediated its interaction with ASH2L (Figure S1G and H). Because ASH2L is a shared subunit by all of the hSET1/MLL-like complexes, we further tested the specificity of the USF1 and hSET1A interaction by analyzing co-precipitated USF1-associated proteins in extracts derived from K562 cells that overexpress Flag-tagged USF1 (Figure S1I). USF1 specifically interacted with hSET1A, but not with the MLL complex (Figure 1F). It was reported that CFP1 is also a component of the hSET1A complex that specifically targets H3K4me3 to CpG islands in ESCs [33]. To test if this component is present in the USF1 associated hSET1A complex, we carried out coimmunoprecipitation assay in Flag-USF1 expressing K562 cells (Figure 1G). Although both USF1 and CFP1 interact with hSET1A, USF1 and CFP1 did not associate with each other (Figure 1G) indicating that the CFP1 component is excluded from the USF1 associated hSET1A complex.

To ascertain that ASH2L mediates USF1 recruitment of the hSET1A core complex, hSET1A core components, hSET1A, WDR5, RBBP5, plus HCF1, were co-expressed in baculovirus-infected insect SF9 cells in the presence or absence of the ASH2L component (Figure 1H, bottom). Reconstitution of the hSET1A complex was verified in SF9 cells (Figure S1J). USF1 pulled down all of the core hSET1A components in the presence but not in the absence of ASH2L (Figure 1H, Top) indicating that ASH2L bridges the association of USF1 with the hSET1A complex.

### Recruitment of hSET1A by USF1 correlates with *HoxB4* gene expression during hematopoiesis

**Figure 2:**
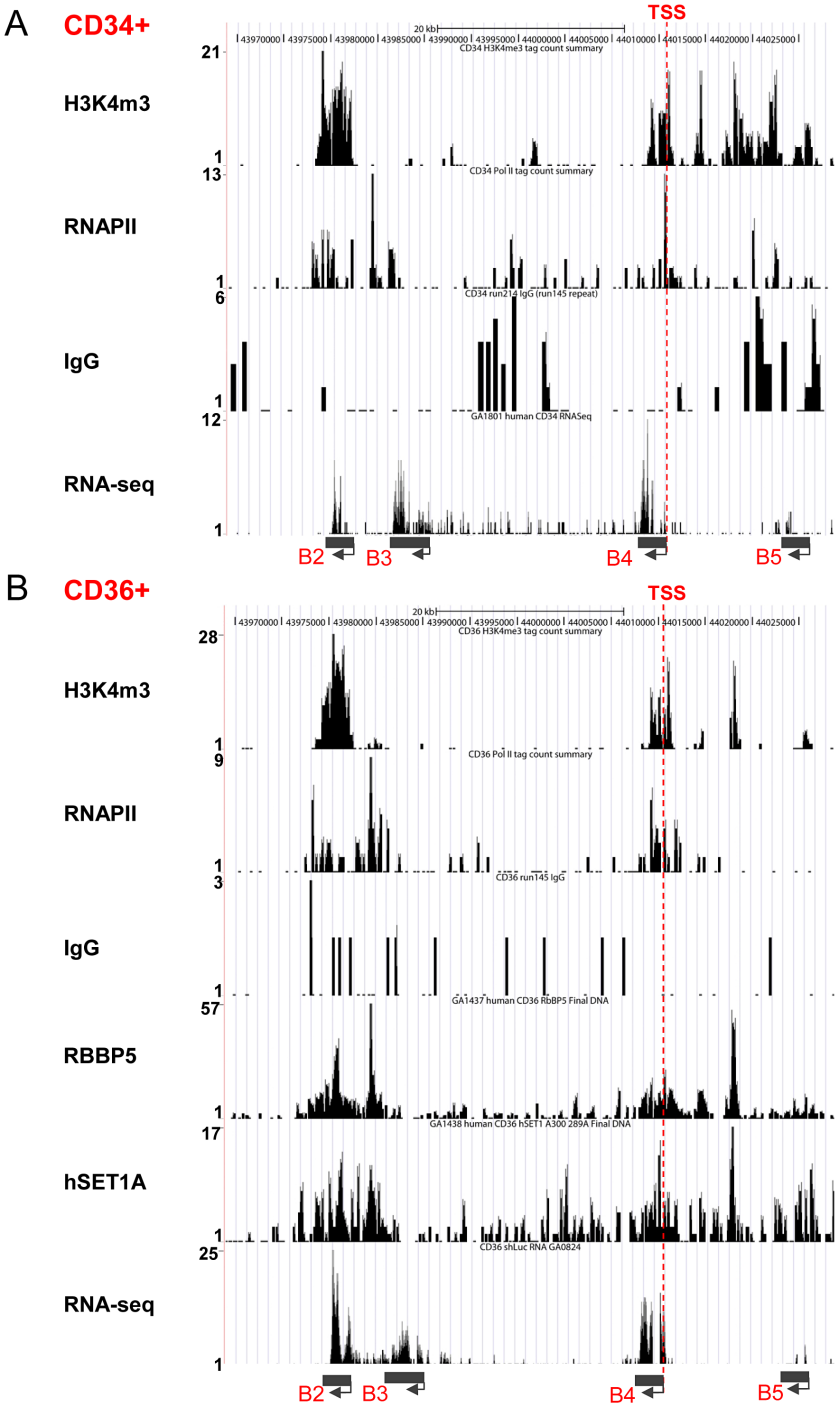
Recruitment of the hSET1A complex and H3K4me3 correlates with expression of the *HoxB4* gene during hematopoiesis. (A) ChIP-seq and RNA-seq analyses of *HoxB4* expression, H3K4me3 enrichment, and RNAPII loading at the anterior *HoxB* locus in CD34^+^ HSCs. (B) ChIP-seq and RNA-seq analyses of *HoxB4* expression, hSET1A recruitment, RBBP5 binding, H3K4me3 enrichment, and RNAPII loading at the anterior *HoxB* locus in CD36^+^ Hematopoietic progenitors.

The hSET1A complex is responsible for H3K4me3 patterns that mark transcriptionally active chromatin. Having implicated hSET1A in hematopoietic transcription of *HoxB4* in K562 cells, we carried out un-biased ChIP-seq and RNA-seq analyses in human primary CD34+ HSCs and CD36+ progenitors to investigate whether targeting hSET1A and its HMT activity are critical for *HoxB4* transcription during hematopoiesis. RNA-seq data revealed that *HoxB4* was actively transcribed in both CD34+ and CD36+ cells (Figure 2A and B). Consistent with the transcription status of the *HoxB4* gene in these cells, H3K4me3 and RNAPII were enriched around the transcription start site (TSS) of the *HoxB4* gene compared to the IgG control which did not bind to the locus (Figure 2A and B). Furthermore, the hSET1A and RBBP5 components of the hSET1A complex were also associated with the TSS (Figure 2B), suggesting that recruitment of the hSET1A complex causes promoter H3K4 methylation and transcriptional activation of *HoxB4* expression in hematopoietic cells. Moreover, the hSET1A complex, H3K4me3, and RNAPII were enriched in the highly transcribed β-*actin* gene but not in the silenced *MyoD1* gene (Figure S2 and S3).

**Figure 3:**
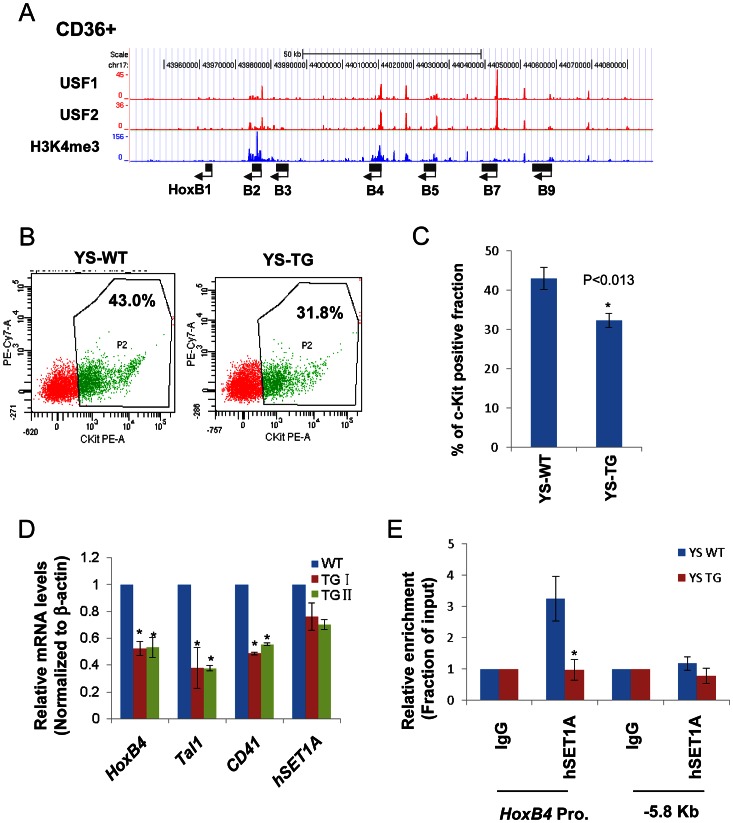
USF1 is responsible for the recruitment of the hSET1A complex and transcriptional activation of *HoxB4* during hematopoiesis. (A) ChIP-seq analyses of USF1, USF2, and H3K4me3 levels at the anterior *HoxB* locus in primary CD36^+^ erythroid cells. (B) Representative FACS analysis of the c-Kit positive primitive HSCs isolated from E9.5 yolk sac comparing WT and AUSF1 transgenic mice. (C) Percentage of the c-Kit positive primitive HSCs isolated from E9.5 yolk sacs of four WT and four AUSF1 transgenic embryos. Data are shown as mean ± SD. * P<0.05. (D) Real-time RT-qPCR analyses of *HoxB4*, *Tal1*, *CD41*, and *hSET1A* mRNA transcript levels in AUSF1 transgenic mice. Data are shown as mean ± SD. * P<0.05. (E)ChIP analysis of the effect of AUSF1 on the recruitment of the hSET1A complex at the *HoxB4* locus in the yolk sac HSCs comparing WT and AUSF1 transgenic embryos. Data are shown as mean ± SD. * P<0.05.

To assess whether the interaction between USF1 and hSET1A is important for *HoxB4* expression during hematopoiesis, we also performed USF1 and USF2 ChIP-seq analyses in CD36+ erythroid precursor cells (Figure 3A). The binding of USF correlated with H3K4me3 patterns at the anterior *HoxB* loci especially at the *HoxB4* transcription start site (TSS; Figure 3A). Next, we examined the effect of USF1 and hSET1A in the regulation of *HoxB4* expression and hematopoiesis using an AUSF1 transgenic mouse model [34]. AUSF1 expressing males die at E11.5–12.5 with severe defects in yolk sac hematopoiesis [34]. The c-Kit positive primitive hematopoietic progenitor cell population was consistently reduced by 24.80% in four AUSF1 transgenic embryos (Figure 3B and C). Reduction of USF1 DNA binding activity in transgenic embryos led to a significant decrease in the expression of *HoxB4*, *Tal1*, as well as CD41 which is the earliest embryonic HS/PC marker [35]–[37], but not hSET1A (Figure 3D). The reduction of *HoxB4* expression was accompanied by a reduction of hSET1A recruitment by 66% at the *HoxB4* promoter region (Figure 3E), suggesting that USF1 guides the hSET1A complex to activate *HoxB4* transcription during primitive hematopoiesis. Taken together, our data demonstrate that USF1 cooperates with the hSET1A complex to regulate hematopoietic-specific *HoxB4* transcription during early embryonic hematopoiesis.

### HS/PC-specific genes including *HoxB4* are repressed by bivalent domains in ESCs and activated by the recruitment of hSET1A upon differentiation

**Figure 4:**
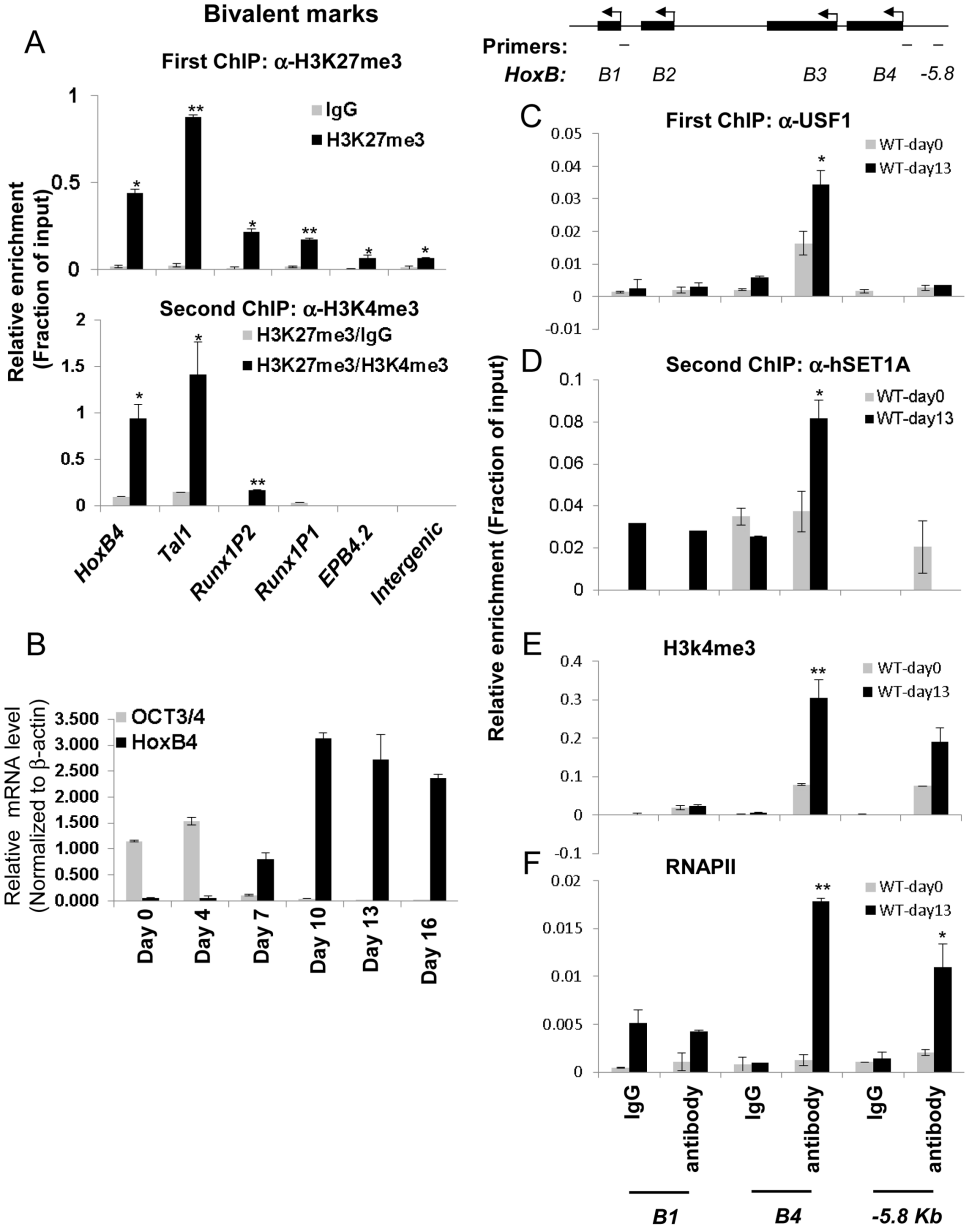
Co-localization of USF1 and the hSET1A complex correlates with the activation of the HSC-specific *HoxB4* gene during hematopoietic differentiation of ESCs.

It has been reported that the ability of ESCs to differentiate into different cell lineages including hematopoietic precursors depends on the balance between self-renewal and cell-fate specification [2]. Lineage commitment-associated genes are modulated in part by bivalent chromatin domains that keep developmental genes in a repressed but poised configuration [3]. To understand the molecular mechanisms governing pluripotent stem cell differentiation into hematopoietic cells, we explored the possibility that HS/PC-specific genes including *HoxB4*, *Tal1*, and *Runx1* are repressed by bivalent chromatin in ESCs and induced by the hSET1A complex during differentiation. A sequential-ChIP analysis was carried out in undifferentiated cells to determine whether H3K4me3 and H3K27me3 co-occupy the promoters of transcription factors associated with early hematopoiesis. Figure 4A shows that in undifferentiated mESCs, *HoxB4* and *Tal1*, which are required for the early onset of hematopoiesis, were marked by both H3K4me3 and H3K27me3 (Figure 4A, bottom, and S4A) while the late erythroid stage gene *EPB4.2* was associated only with H3K27me3 (Figure 4A). The *Runx1* gene is also critical for HS/PC function and contains two promoters [38]. Interestingly, its *P1* promoter, which is activated and required for late myeloid lineage differentiation, was associated with high levels of H3K27me3 alone (Figure 4A). In contrast, the *P2* promoter, which is exclusively active at the HSC stage [38], was marked by bivalent chromatin domains (Figure 4A, bottom). Thus, our data indicate that the promoters of HS/PC-associated genes are repressed by bivalent domains in undifferentiated ES cells.

Given that ectopic activation of the *HoxB4* gene in ESCs leads to hematopoietic fate specification [20]–[25], we further reasoned that the guided recruitment of hSET1A to the *HoxB4* locus by USF1 may initiate *HoxB4* transcription and hematopoietic differentiation of ESCs. To test this possibility, ESCs were induced to differentiate into hematopoietic cells by addition of stem cell factor (SCF), IL3, and IL6 (Figure S4B). Under these conditions, ESCs differentiated into hematopoietic cells and expressed lineage markers and hematopoietic specific transcription factors (Figure S4C–F). Time course ChIP-qPCR analyses indicated that, although USF1 and hSET1A levels were not increased during differentiation (Figure S4D), USF1 binding, hSET1A recruitment, and H3K4me3 levels were increased and remained at high levels at the *HoxB4* promoter after day 10 of differentiation (Figure S5A–C). As expected, expression of ESC-specific transcription factor *Oct4* was dramatically reduced while hematopoietic-associated *HoxB4* was gradually increased upon differentiation (Figure 4B). *HoxB4* expression reached its peak on day 10 of differentiation and remained at a high level (Figure 4B). Consistent with the *HoxB4* expression pattern, the recruitment of USF1 and hSET1A to the *HoxB4* promoter increased upon differentiation (Figure 4C, 4D, and S5A–C). In contrast, MLL1 and MLL2 did not bind to the *HoxB4* promoter in either K562 or ESCs (Figure S5D and data not shown). Indeed, sequential ChIP analyses showed that USF1 and hSET1A co-occupied the *HoxB4* promoter only in hematopoietic cells differentiated from ESCs, but not in undifferentiated ESCs (Figure 4D). The recruitment of hSETA1 was coincident with a significant increase of H3K4me3 at the active *HoxB4* promoter (Figure 4E). Consistent with hSET1A recruitment and H3K4me3 patterns, the binding of RNAPII was also significantly elevated by 8.2 fold at the *HoxB4* promoter upon induction compared to undifferentiated ESCs (Figure 4F). These data suggest a functional relationship between hSET1A recruitment and activation of hematopoietic lineage-associated genes during ESC differentiation.

### USF1 regulates ESC pluripotency through controlling mesoderm and lineage specific differentiation

**Figure 5:**
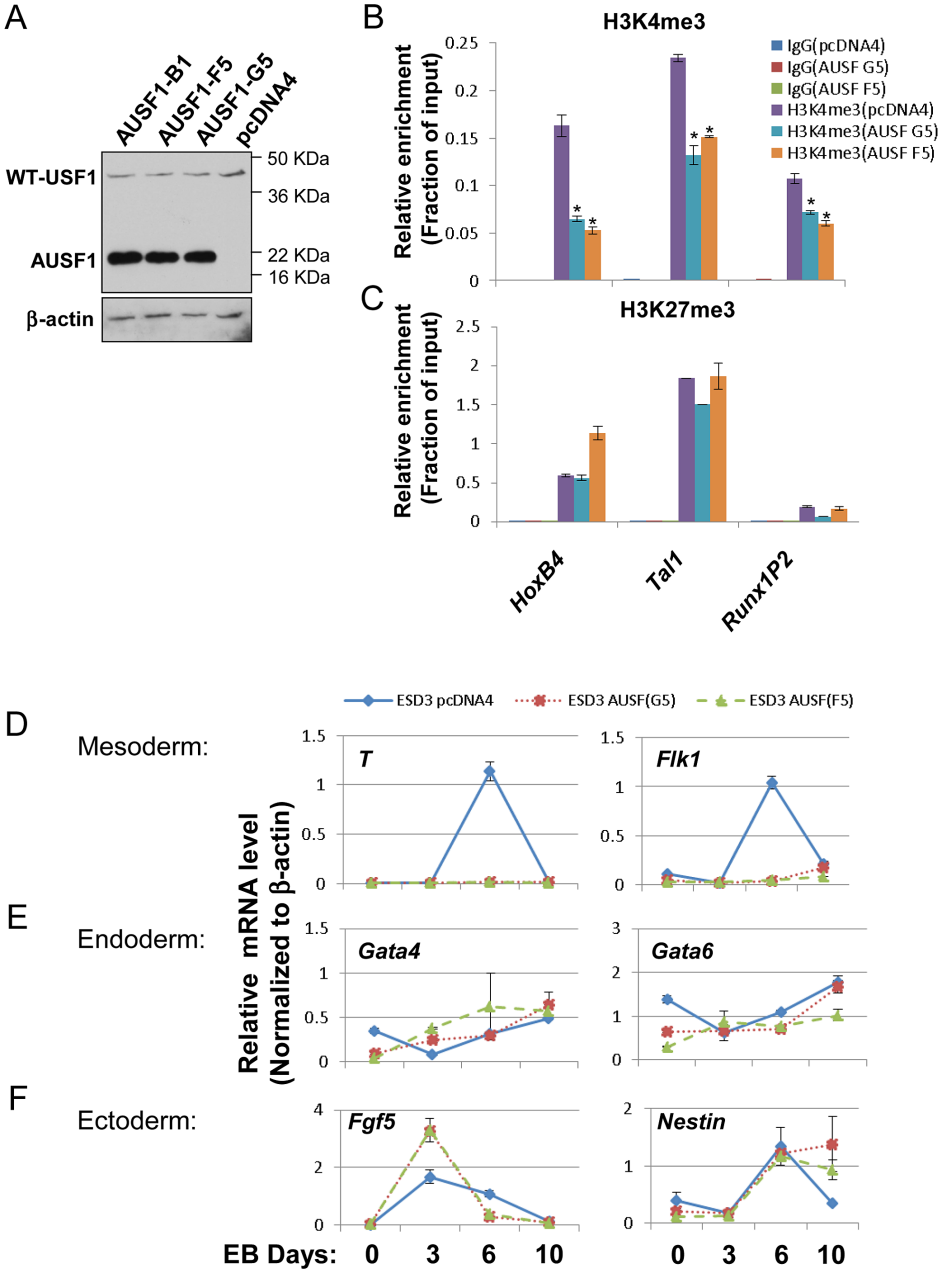
USF1 regulates ESC pluripotency by specifically controlling mesoderm differentiation. (A) Immunoblot assay showing the levels of endogenous USF1 and ectopically expressed AUSF1 in pcDNA transfected and AUSF1 expressing mES cells. (B) ChIP analyses of H3K4m3 levels at the HSC-associated *HoxB4*, *TAL1*, and *Runx1* genes comparing the pcDNA transfected control and the dominant negative AUSF1 overexpressing ESCs. Data are shown as mean ± SD. * P<0.05 and ** P<0.01. (C) ChIP analyses of H3K27m3 levels at the HSC-associated *HoxB4*, *TAL1*, and *Runx1* genes comparing the pcDNA transfected control and the dominant negative AUSF1 overexpressing ESCs. Data are shown as mean ± SD. * P<0.05 and ** P<0.01. (E) to (F)Time course qRT-PCR analyses of the expression levels of mesoderm (D), endoderm (E), and ectoderm (F) markers in pcDNA control and two AUSF1 overexpressing clones upon withdrawal of LIF.

Having shown that USF1 activity is important for *HoxB4* expression in differentiation of ES cells (Figure 4C–F and S5A–C), we next examined the biological effect of USF1 on stem cell pluripotency and differentiation by enforced expression in ES cells of AUSF1, a dominant negative USF1 mutant that competes with endogenous USF for DNA-binding (Figure 5A). Disruption of USF1 DNA binding activity affected neither expression of stemness genes *Oct4* and *Nanog* (Figure S6A) nor the self-renewal ability of ESCs as examined by AP staining (Figure S6B). In addition, we tested the relationship of enforced AUSF1 expression and changes in bivalent H3K4 and H3K27 trimethylation patterns at HS/PC-specific genes in ESCs. Suppression of USF DNA-binding activity in ESCs resulted in a specific inhibition of H3K4me3 levels within HS/PC-associated bivalent chromatin marks in the *HoxB4*, *Tal1*, and *Runx1P2* gene loci (Figure 5B), but did not reduce the H3K27me3 levels at these genes (Figure 5C). Interestingly, AUSF1 embryoid bodies (EBs) appeared smaller compared to those harboring the vector control (Figure S6C), suggesting that loss of USF activity compromises lineage differentiation programs. We next examined the expression of endoderm, mesoderm, and ectoderm markers in both control and AUSF1 EBs (Figure 5D–F). To our surprise, inhibition of USF1 DNA binding activity in early embryonic development resulted in a specific reduction of primitive mesoderm markers, *Brachyury (T)* and *FLK1*, with only minor effects on endoderm and ectoderm markers, *Gata4*, *Gata6*, *Fgf5*, and *nestin* (Figure 5D–F). Further analysis indicated that USF1 does not directly regulate both *T* and *FLK1* (Figure S6D). Several mesoderm markers, *Mesp1*, *Snail2*, *Eomes*, and *Nkx2-5*, were also significantly inhibited by ectopic expression of AUSF1 (Figure S6E). The effect of USF1 in EB differentiation was biased toward the mesoderm which can differentiate to hematopoietic lineages. This is consistent with the disruption of H3K4me3 levels at HS/PC-associated chromatin domains by enforced expression of AUSF1.

**Figure 6:**
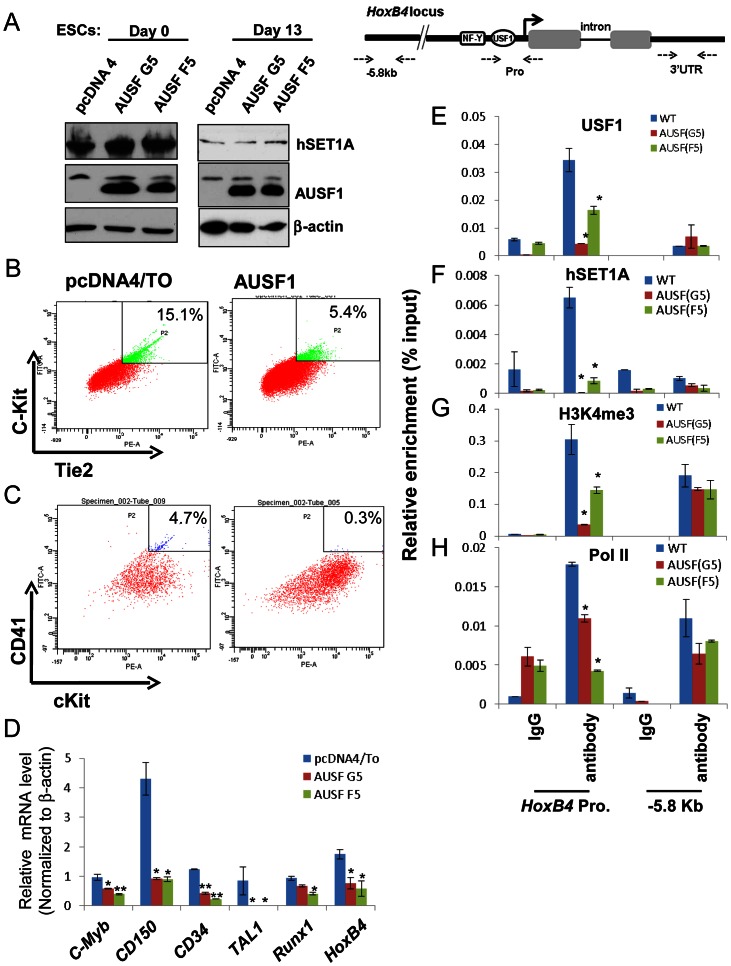
USF1 regulates ESC fate and hematopoietic differentiation by modulating bivalent domains of HS/PC-specific genes. (A) Western blotting assay of hSET1A levels in undifferentiated (day 0) and differentiated (day 13) ESCs comparing pcDNA vector transfected control and AUSF1 overexpressing cells. (B) FACS analysis of c-Kit and Tie2 expressing hemogenic endothelium cell population in pcDNA tranfected control and AUSF1 overexpressing ESCs upon hematopoietic differentiation at day 6. (C) FACS analysis of CD41 and c-Kit expressing early hematopoietic stem and progenitor population in pcDNA transfected control and AUSF1 overexpressing ESCs upon hematopoietic differentiation at day 10. (D) Real-time RT-qPCR analysis of hematopoietic markers and transcription factors, *c-Myb*, *CD150*, *CD34*, *Tal1*, *Runx1*, and *HoxB4* mRNA transcript levels upon hematopoietic differentiation comparing pcDNA transfected control and two AUSF1 overexpressing ES clones. Data are shown as mean ± SD. * P<0.05. (E) to (H). ChIP analyses of USF1 binding (E), hSET1A recruitment (F), H3K4me3 enrichment (G), and RNAPII loading (H) at the *HoxB4* promoter in AUSF1 overexpressing ES clones. Data are shown as mean ± SD. * P<0.05.

The mouse mesoderm derived aorta-gonad mesonephros (AGM) region is considered to be the first site of hematopoiesis [39] and hematopoietic potential is segregated within the Flk1^+^ compartment in the murine EB system [40], [41]. The hemogenic endothelium stage cells (Tie2^hi^c-Kit^+^ population) developed from hemangioblast give rise to HS/PCs at the onset of hematopoiesis [37], [42]–[45]. AUSF1 specifically inhibited Flk1 expression in the mesoderm (Figure 5) suggesting that USF1 may be involved during early hematopoietic commitment steps. We next explored the effect of AUSF1 expression on cytokine induced hematopoietic differentiation of ES cells (Figure S4B). Ectopic expression of AUSF1 did not affect co-activator hSET1A expression in undifferentiated or differentiated ESCs (Figure 6A). However, it specifically inhibited differentiation of hematopoietic progenitors in three independent AUSF1 ESC clones (Figure 6B, C, and S7A). Compared to cells harboring the pcDNA vector control, cells expressing AUSF exhibited a reduction in c-Kit and Tie2 double positive hemogenic endothelium cell population by 64% (down to 5.4% from 15.1%; Figure 6B). These cells represent hematopoietic potential at the onset of hematopoiesis [37], [43]. Furthermore, suppression of USF1 activity significantly reduced the number of CD41^+^c-Kit^+^ HS/PCs (down from 4.7% to 0.3%) (Figure 6C) and reduced Sca1^+^c-Kit^+^ hematopoietic cells by 75% (Figure S7A and 7B). In addition to the inhibition of hematopoietic cell populations, the HS/PC markers and transcription factors associated with the onset of hematopoiesis such as *HoxB4*, *Tal1*, and *Runx1* were downregulated by the inhibition of USF1 activity (Figure 6D). AUSF1 only dimerized with wt USF1 but not with TAL1 (Figure S7C), as expected [46]. Therefore, AUSF1 did not interfere with the DNA binding activity of hematopoietic specific bHLH transcription factor TAL1 (Figure S7C and D). Thus, the data show that USF1 is critical for the initial expression of hematopoietic transcription factors and markers.

### Recruitment of hSET1A by USF1 is critical for hematopoietic expression of the *HoxB4* gene

To further elucidate the underlying mechanism by which USF1 regulates hematopoietic associated *HoxB4* transcription, USF1 binding, hSET1A recruitment, H3K4me3 levels, and RNAPII loading at the *HoxB4* locus were examined in hematopoietic cells differentiated (days 13) from ESCs either harboring a pCDNA4 vector or expressing AUSF1. Enforced expression of AUSF1 suppressed *HoxB4* transcription by an average of 61.8% compared to the vector control (Figure 6D). In contrast, AUSF1 did not affect hSET1A expression (Figure 6A and S6A). Consistent with the decrease in *HoxB4* transcription (Figure 6D), AUSF1 expression eliminated the binding of USF1 and consequently the recruitment of hSET1A at the *HoxB4* promoter (Figure 6E and F). The elimination of hSET1A recruitment was accompanied by a dramatic loss of H3K4me3 levels (reduced by 66.5% on average) and a decrease in Pol II binding at the *HoxB4* promoter (Figure 6G and H). These data reveal that USF1 activates lineage-specific transcription by recruiting the hSET1A complex to hematopoietic regulatory elements.

### hSET1A is required for ESC lineage differentiation, but not self-renewal

**Figure 7:**
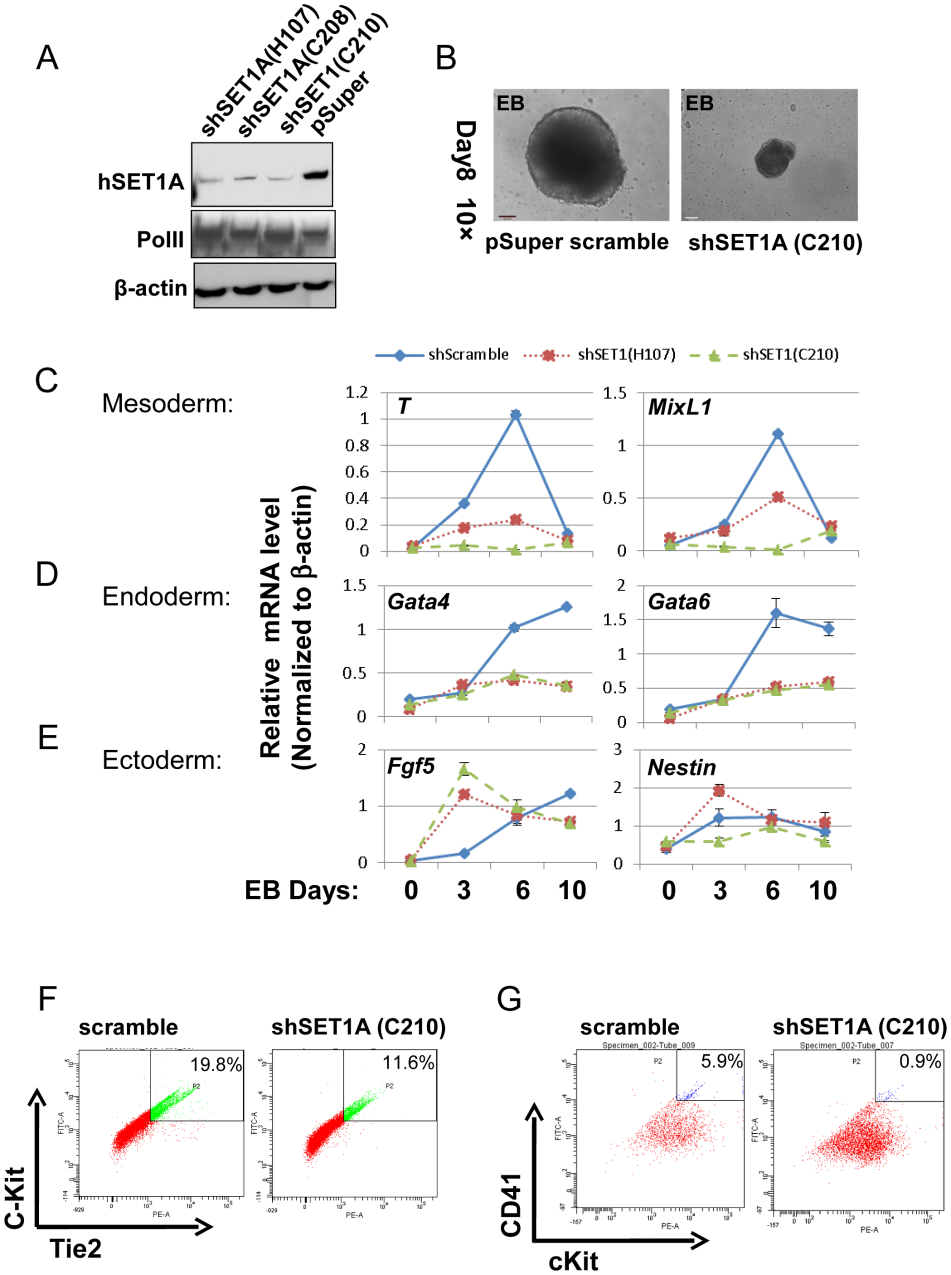
Effect of hSET1A depletion on ESC mesoderm differentiation and hematopoiesis. (A) Western blotting analysis of hSET1A protein levels in ESCs harboring scramble control or hSET1A specific shRNAs. (B) Hematopoietic differentiation assay. The scramble control or hSET1A KD ESCs were cultured in suspension without LIF to induce EB formation for 4 days and then cultured in the presence of SCF to induce hematopoietic differentiation for another 4 days. Shown are EBs after 8 days in culture. Scale bar, 100 µm. (C) to (E) Time course qRT-PCR analyses of the levels of mesoderm (C), endoderm (D), and ectoderm (E) markers in the scramble control and two hSET1A KD clones upon withdrawal of LIF. (F) FACS analysis of c-Kit and Tie2 expressing hemogenic endothelium population in the scramble control and hSET1A KD ESCs upon hematopoietic differentiation at day 6. (G) FACS analysis of CD41 and c-Kit expressing early hematopoietic stem and progenitor population in the scramble control and hSET1A KD ESCs upon hematopoietic differentiation at day 10.

The above data indicate that the activity of USF1 during mesoderm differentiation and subsequent transcription activation of *HoxB4* correlates with its ability to recruit the hSET1A complex (Figure 5 and 6D–G). To ascertain whether hSET1A, but not MLL1, is required for USF1-mediated transcription activation during differentiation, hSET1A was silenced in ES cells by retroviral-mediated shRNA targeting (Figure 7A). Compared to the pSuper scramble control, three individual ES clones showed a substantial decrease in hSET1A expression (Figure 7A). ESCs transduced with retroviruses harboring the pSuper scramble control or the hSET1A-specific shRNAs were differentiated into embryoid bodies (EBs) by withdrawal of LIF (Figure S4B). Similar to the ectopic expression of AUSF1, downregulation of hSET1A did not alter ESC self-renewal properties and expression of the *Oct4* and *Nanog* genes (Figure S8A and S8B), but impaired EB size (Figure 7B). Furthermore, we examined the expression levels of three germ layer lineage markers in the scramble and hSET1A KD EBs by qRT-PCR (Figure 7C–E). Both mesoderm and endoderm markers were largely abolished in the hSET1A KD EBs. However, ectoderm markers were not suppressed by the hSET1A KD.

**Figure 8:**
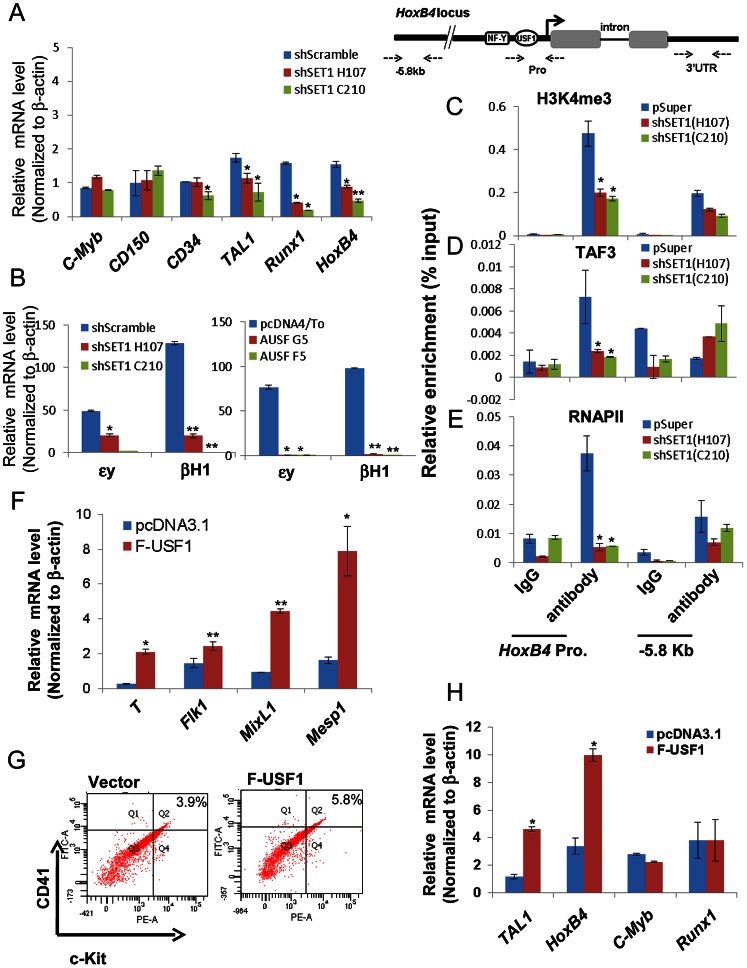
USF1 and hSET1A together activate expression of transcription factors associated with early onset of hematopoiesis during ESC differentiation. (A) Real-time RT-qPCR analysis of hematopoietic markers and transcription factors, *c-Myb*, *CD150*, *CD34*, *Tal1*, *Runx1*, and *HoxB4* mRNA transcript levels upon hematopoietic differentiation comparing the scramble control and two hSET1A KD ES clones. Data are shown as mean ± SD. * P<0.05. (B) Real-time RT-qPCR analysis of primitive hematopoietic markers, εy and βH1, upon hematopoietic differentiation comparing control and hSET1A KD (Left) or AUSF1 overexpressing (Right) ESCs. (C) to (E) ChIP analyses of H3K4me3 (C), TAF3 (D), and RNAPII (E) binding at the *HoxB4* promoter after hSET1A KD upon hematopoietic differentiation at day 10. Data are shown as mean ± SD. * P<0.05. (F) Real-time RT-qPCR analysis of mesoderm markers in the vector control and Flag-USF1 overexpressing ES cells upon withdrawal of LIF. (G) FACS analysis of CD41 and c-Kit expressing early hematopoietic stem and progenitor population in control and Flag-USF1 expressing ESCs upon hematopoietic differentiation at day 10. (H) Real-time RT-qPCR analysis of transcription factors association with early onset of hematopoiesis in the vector control and Flag-USF1 overexpressing ES cells upon withdrawal of LIF.

Further analyses of the hematopoietic population derived from EBs following a switch to suspension culture with hematopoietic differentiation medium for 6 (for Tie2^+^c-Kit^+^ hemogenic endothelium population) or 10 (for CD41^+^c-Kit^+^ population) days revealed that similar to the effect of AUSF1, the Tie-2^+^c-Kit^+^ hemogenic endothelium cell population, which is prone to hematopoiesis, was significantly decreased by 41.4% in the hSET1A KD cells (Figure 7F). In addition, both CD41^+^c-Kit^+^ as well as Sca-1^+^c-Kit^+^ hematopoietic cell populations were reduced by 85% and 43.5% in the hSET1A KD cells compared to cells expressing the scrambled shRNA (Figure 7G, S8C, and S8D), respectively. Expression of transcription factors associated with the onset of hematopoiesis was also inhibited by the hSET1A KD (Figure 8A). Interestingly, in both AUSF1 and hSET1A KD EBs primitive hematopoiesis markers ε*y* and *βH1* were significantly downregulated (Figure 8B) indicating that USF1 and the hSET1A complex in part work together to regulate early hematopoiesis.

Transcription of the *HoεxB4* gene is inhibited by both expression of AUSF1 and hSET1A KD (Figure 6D and 8A). Disruption of the hSET1A complex may lead to a loss of H3K4 methylation that subsequently impairs recruitment of the RNAPII transcription pre-initiation complex to USF1 target genes such as *HoxB4* gene. To test this hypothesis, ChIP-qPCR was performed in the pSuper scramble control and hSET1A KD ESC-derived hematopoietic cells. H3K4me3 levels at the *HoxB4* promoter were significantly reduced by 55% and 58% in two selected hSET1A depleted clones, respectively (Figure 8C). TAF3 is a component of the TFIID complex and specifically recognizes the H3K4me3 mark at promoters, thereby transmitting the histone methylation signal to the recruitment of RNAPII [47]. Thus, we tested whether reduction of H3K4me3 at the *HoxB4* promoter by hSET1A KD results in subsequent decreased recruitment of TAF3 or RNAPII. Consistent with the observed changes in H3K4me3 patterns, levels of *HoxB4* promoter bound TAF3 and RNAPII were also reduced 66% and 85% in hSET1A depleted clones, respectively (Figure 8D and 8E). This is consistent with a previous report demonstrating that the PHD domain of TAF3 recognizes the H3K4me3 mark [47]. Taken together, our data demonstrate that recruitment of the hSET1A complex and its HMT activity plays a critical role in activating a lineage-specific transcription program during differentiation of ESCs.

### Ectopic expression of USF1 in ES cells enhances mesoderm development and lineage differentiation

Finally, we tested whether USF1 induces lineage differentiation by inducing transcription factors associated with the early onset of hematopoiesis. Flag-tagged USF1 was stably expressed in murine ES cells (Figure S9A).We used RT-qPCR to survey three lineage markers in control and USF1 overexpressing EBs upon withdrawal of LIF for 6 days (Figure 8F). As expected, only mesoderm markers (Figure 8F), but not endoderm or ectoderm markers (Figure S9B), were upregulated by overexpression of USF1 supporting the notion that USF1 plays an important role in mesoderm development.

To further examine the effect of USF1 overexpression on hematopoietic differentiation, both control and USF1 expressing EBs were collected and induced with hematopoietic cytokines to differentiate these cells along the hematopoietic lineage (Figure S4B). FACS analysis revealed that USF1 overexpression increased the CD41^+^c-Kit^+^ hematopoietic cell population from three independent experiments (Figure 8G and S9C). The *CD41^+^c-Kit^+^* population was increased by 42.8% in USF1 expressing cells compared to cells harboring the vector control (from 3.9% in control cells to 5.57% in USF1 overexpressing cells) (Figure S9D). Although c-Kit is present in all hematopoietic cell populations, only the earliest embryonic HSCs express CD41 [35]. Consistent with the role of CD41 in embryonic hematopoiesis, both primitive hematopoietic markers, ε*y* and *βH1*, were significantly upregulated in the USF1 overexpressing ES cell clones (Figure S9E). The levels of *Tal1* and *HoxB4* that associate with the early onset of hematopoiesis were also significantly stimulated by 2.8 and 3 fold in cells overexpressing USF1, respectively (Figure 8H). Thus, our data suggest an important role of USF1 and hSET1A in directing lineage fate determination during ESC differentiation.

## Discussion

The dynamic balance of H3K4me3 and H3K27me3 at genes involved in the commitment and development of multiple lineages is thought to play a critical role in maintaining the differentiation potential of ESCs [1], [3]–[5]. However, it remains unknown how chromatin modifying enzymes that catalyze these modifications are targeted to lineage-specific genes to allow lineage commitment of ESCs. Do variations in the enzymatic subunit compositions of the MLL1, MLL2, MLL3, MLL4, hSET1B, or hSET1A complexes play different roles in ESC self-renewal versus differentiation? It has been suggested that hSET1A and hSET1B play non-redundant roles in the regulation of chromatin structure and gene expression [48]. We show that the hSET1A HMT complex is essential for ESC differentiation, but not self-renewal. We demonstrate that hSET1A is recruited to hematopoietic associated genes by transcription factor USF1 through its interaction with ASH2L, a core component of hSET1/MLL complexes. By doing so, USF1 and hSET1A cooperatively shift the balance of bivalent chromatin modifications to H3K4me3, thereby driving lineage differentiation. In this regard, we provide evidence demonstrating that the DNA-sequence specific transcription factor USF1 initiates and enforces hematopoietic associated *HoxB4* expression in differentiating ESCs by guiding the hSET1A complex and corresponding H3K4me3 to the *HoxB4* promoter. H3K4me3 is then recognized and bound by TAF3 and RNAPII in differentiating ESCs (Figure 6 and 8). It is particularly interesting that TAF3 also controls ESC lineage commitment by regulating the balance of transcription programs involved in ESC pluripotency and commitment [49].

Select components of the hSET1/MLL complexes have been implicated in both regulating stem cell self-renewal and lineage-fate commitment [11], [12]. H3K4me3 appears to be required for maintaining high level expression of pluripotency associated genes and for transcriptional induction of lineage-specific regulators. The differential roles of hSET1/MLL complexes in ESC commitment and self-renewal may rely on the specific loci to which the different complexes are recruited. All of the hSET1/MLL complexes consist of shared structural core subunits but vary in their specific enzymatic and several additional subunits [50], [51]. In this regard, it is interesting to note that transcription factor USF1, which is critically involved in activating the *HoxB4* gene in the hematopoietic lineage, is associated with the hSET1A complex, but not with the MLL complex. Furthermore, MLL1 is required for the activation of other anterior *HoxB* genes but not *HoxB4* in acute myeloid leukemia, and deletion of MLL1 does not affect *HoxB4* transcription [17], [26]. Thus, we propose that the different hSET1/MLL complexes play unique roles during differentiation and development. Our data demonstrate that USF1 specifically interacts with the hSET1A complex via the ASH2L subunit, which is also common to the other MLL complexes. Thus, there must be additional determinants for this interaction, likely complex-specific subunits, that prevent stable USF1 interactions with other (e.g., MLL1) complexes.

Apart from the ESC core transcriptional network of genes (*Oct4*, *Nanog*, and *Sox2*) that regulate self-renewal, factors controlling transcription programs that guide lineage commitment are largely unknown. In order to simultaneously maintain both stem cell identity and differentiation potential, ES cells possess unique bivalent chromatin domains at gene loci expressing developmental regulators [3]–[5]. Alteration of either H3K4 methylation or H3K27 methylation may perturb commitment of ESCs. USF1 controls cell fate specification through regulation of H3K4 methylation marks at lineage-specific genes although USF1 does not recruit hSET1A to these loci in undifferentiated ESCs. Interestingly, USF1 can function as a chromatin barrier to maintain active histone modifications within chromatin domains in hematopoietic cells [31], [52], which may be involved in protecting HS/PC-associated bivalent genes in undifferentiated ESCs and ascertain that these genes are primed to be induced in response to differentiation. In this case, it is interesting to note that USF1 also interacts with another H3K4 HMT, SET7/9 which may contribute to the barrier activity of chicken HS4 [52]. In agreement with this notion, the ectopic expression of dominant negative AUSF1 in ESCs and in transgenic mice inhibits differentiation of mesoderm and further hematopoietic lineages (Figures 3, 5 and 6).

Although USF1 is a ubiquitous transcription factor/chromatin barrier protein, it has been implicated in hematopoiesis and β-globin gene regulation through association with a variety of cofactors that include the hSET1A complex [31], [34] (this report). Thus, an interesting question is what underlies selective differentiation bias toward hematopoiesis. The most plausible explanation is that the disruption of USF1 activity broadly impairs gene expression patterns especially those critical for mesoderm development. Some of the USF1 targets are transcription factors required for hematopoietic commitment and differentiation. Consistent with this mechanism, both inhibition of USF1 DNA binding and depletion of hSET1A led to a decrease in expression levels of transcription factors associated with the onset of hematopoiesis, *HoxB4*, *Tal1*, and *Runx1* (Figure 6 and 8). In particular, the homeodomain gene *HoxB4* has been shown to promote engraftment of murine bone marrow HSCs and enhance self-renewal of HS/PCs from human cord blood [20], [21], [23]–[25]. Involvement of USF1 in *HoxB4* activation [27] suggests a role of USF in regulating hematopoietic differentiation. Ectopic expression of USF1 enhances formation of the CD41^+^c-Kit^+^ HS/PC subpopulation and activates expression of hematopoietic associated *HoxB4* and *Tal1* genes supporting its role in hematopoiesis (Figure 8). Interestingly, overexpression of NF-Ya, a transcription factor that forms a complex with USF and binds to the *HoxB4* promoter [30], also specifically promoted hematopoiesis of hESCs (personal communication with Dr. Stephen Emerson). It is possible that NF-Ya cooperates with USF to activate the *HoxB4* promoter by dramatically increasing the affinity of the protein complex to the *HoxB4* promoter chromatin in hematopoietic cells [27],[30]. Finally, induced recruitment of the hSET1A HMT complex by USF1 to the *HoxB4* promoter upon hematopoietic differentiation (Figure 4) likely leads to alterations in chromatin structure and stabilize transcription complexes at hematopoietic promoters.

In mammals, *HoxB4* is expressed in the stem cell fraction of the bone marrow. Ectopic expression of the *HoxB4* gene in bone marrow hematopoietic cells leads to a dramatic expansion and increased self-renewal of HSCs [21], [23], [24]. Furthermore, *HoxB4* expression promotes the transition from embryonic stem cells into definitive HSCs with increased long-term engraftment potential [20], [25], suggesting that *HoxB4* is an early hematopoietic regulator associated with hematopoietic fate specification of ESCs. Our data reveal that *HoxB4* and other hematopoietic associated genes are modulated by bivalent chromatin domains (Figure 3). The main action of USF1 and the co-regulator hSET1A during differentiation of ESCs is to ascertain activation of the *HoxB4* gene as well as other hematopoietic regulators that shifts the decision of differentiation versus self-renewal to reinforce hematopoietic commitment. However, it still remains to be determined whether *HoxB4* is sufficient for driving ESC differentiation. It is reported that *HoxB4* deficient mice exhibit mild reduction in progenitors and stem cells in fetal liver and bone marrow [53]. In contrast, mice deficient in both HoxB3 and HoxB4 or compound deletion of Hoxa9/HoxB3/HoxB4 display severe hematopoietic defects with a marked decrease in HSC regeneration and proliferation [54], [55] suggesting that other *Hox* genes may compensate for the loss of *HoxB4*. Nevertheless, the data presented here support the notion that activation of *HoxB4* confers HSC expansion by coordinating the stem cell response to commitment and differentiation signals [20], [22], [25].

## Materials and Methods

### Cell culture, plasmid constructs, AUSF1 transgenic mice, and siRNA mediated KD

K562 cells were cultured in RPMI1640 media supplemented with 10% fetal bovine serum (FBS). Murine ESCs were maintained at a density between 1×10^5^ and 5×10^5^ cells/ml in 5% CO2 at 37°C as described [56]. Constructs used for shRNA expression were generated by subcloning shRNA oliginucleotides into the pSuper.retro.puro vector following the manufacturer's instruction (Oligoengine). Infectious viruses were produced in PhoenixA package cells using calcium phosphate transfection; supernatant was collected after 48 hrs post-transfection to infect cells, and cells were selected by puromycin resistance.

The AUSF1 transgenic mice have been described previously [34]. All animal experiments were approved by the IACUC committee and conform to the regulatory guidelines. The pcDNA4-AUSF1 expressing construct was described previously [31]. The pcDNA3.1-Flag-USF1 expressing vector was cloned by fusing Flag tag to the N-terminal of human USF1 cDNA. ESCs were transfected with these plasmids using Lipofectamine 2000 reagent (Invitrogen) and selected with 400 ng/ml Zeocin for AUSF1 and with 500 µg/ml G418 for Flag-tagged USF1 (Invitrogen). For luciferase reporter assays, K562 cells were transfected with a pREP4-hHoxB4-luc reporter, an expression vector for USF1, AUSF1, or siRNAs targeting individual components of the hSET1A complex, hSET1A, HCF1, or ASH2L. A CMV-driven renilla luciferase plasmid was used as transfection control. Transfected cells were cultured for 48 hrs and subjected to luciferase and ChIP assays.

### *In vitro* differentiation of ESCs

*In vitro* hematopoietic differentiation of ESCs was performed as described previously [56] with minor modifications (Figure S4B). Briefly, mESCs were dispersed into single cell suspension by trypsinization with 0.05% trypsin and resuspended in EB media (IMDM supplemented with 15% FBS, 180 mg/ml transferrin, 4.5×10^−4^ M (MTG), 50 ng/ml ascorbic acid, and 1% penicillin/streptomycin) with 5×10^4^ cells/ml. 20 µl of a drop containing 1000 ESCs were seeded on the cover of the tissue culture dish by the hanging drop method. The EBs were collected for suspension culture in polyHEMA-coated dishes on day 3. The EBs were then supplemented with 40 ng/ml SCF on day 4. Fresh media containing 40 ng/ml mSCF, 20 ng/ml IL3, and IL6 (Peprotech) was replaced on day 7 and then changed with fresh media every two days. The EBs were harvested at different time points, and single cell suspensions were prepared for FACS analysis, RNA extraction, and ChIP.

### Recombinant protein purification, GST pull-down assay, and co-immunoprecipitation

FLAG tagged cDNAs encoding hSET1A, ASH2L, WDR5, RBBP5 and HCF1 were cloned into the pFastBac1 vector as described [14] and proteins were expressed in SF9 cells using the Bac-to-Bac Baculovirus Expression System (Invitrogen). Flag tagged fusion proteins were purified by FLAG immunoaffinity purification as described [14]. To reconstitute the hSET1A core complex, SF9 cells were co-infected with baculoviruses expressing FLAG-tagged hSET1A, ASH2L, RBBP5, WDR5, and HCF1. The GST-pull down assay was carried out as described [31]. Briefly, equal amounts of GST-USF1 fusion protein was incubated with reconstituted hSET1A core complex or individual components of the complex in 500 µl binding buffer (PBS, 10% glycerol, 1% Triton X-100, 1 mM EDTA, 1 mM DTT) at RT for 1 hour and proteins were captured by glutathione beads preconjugated with GST-USF1. Captured proteins were washed three times and detected by western blotting (WB).

Immunoprecipitation was carried out as described previously [31]. USF1 (H-86), MLLn, and MLLc antibodies were purchased from Santa Cruz Biotechnology (Santa Cruz, CA). hSET1A (A300-289A, A300-290A), RbBP5 (A300-109A), ASH2L (A300-489A), HCF1 (A301-399A), and TAF3 antibodies were from Bethyl Laboratories (Montgomery, TX). Mouse FLAG antibody was bought from Sigma.

### FACS analysis of hematopoietic precursors

Single cell suspensions obtained from E10.5 yolk sacs of the AUSF1 embryos treated with collagenase (Stem Cell Technologies) was subjected to FACS analysis using antibody against CD117 PE (c-Kit) (BD Biosciences). Single cell suspension obtained from differentiated EBs was subjected to FACS analysis using antibodies against CD117 PE (c-Kit), Sca-1 PE-cy7, CD41 FIFC (BD Biosciences), CD117 FIFC, or Tie2 PE (eBioscience). Briefly, cells were resuspended in PBS containing 2% FBS, passed through a 70-µm cell strainer, and incubated on ice with indicated antibodies for 30 min. After a series of washes to remove unbound antibodies, cells were subjected to FACS using a BD LSRII system (BD Biosciences).

### RT-qPCR and quantitative PCR

Total RNA was prepared by using the RNeasy mini isolation kit according to the manufacturer's instruction (Qiagen, MD, USA). 1 µg RNA was reverse transcribed by using the Superscript II reverse Transcriptase (Invitrogen). cDNA was analyzed by real-time PCR (qRT-PCR) using a CFX 96 real time PCR Detection System (Bio-Rad). Primer sequences are listed in the Supplemental Information (Table S1).

### Chromatin immunoprecipitation (ChIP), Micro ChIP, and sequential ChIP

ChIP assays were performed as described previously [31] using antibodies specific for transcription factors, modifying enzymes, and various histone modifications. Antibodies against RNAPII, H3K4me3 and H3K27me3 were purchased from Millipore (Millipore). Other antibodies were described above. The relative enrichment was determined by the following equation: 2*^Ct(IP)−Ct(Ref)^*. In addition, Micro-ChIP was used to analyze EBs (1×10^4^ cells/IP) using Dynal bead conjugated protein A or protein G (Invitrogen).

The sequential ChIP assays were carried out as described previously [32] with minor modifications. Briefly, chromatin prepared from 3×10^6^ cells was first immunoprecipitated with USF1 or H3K27me3 antibody. The USF1 or H3K27me3 selected chromatin complexes were eluted, dialyzed, and subsequently immunoprecipitated with hSET1A or H3K4me3 antibody, respectively. The bound protein-DNA complexes were reverse cross-linked, purified, and analyzed by qPCR.

### ChIP-Seq and RNA-seq

Primary human CD34+ cells were isolated and differentiated to CD36+ cells as described [10]. ChIP-Seq and RNA-seq assays were performed as outlined previously [10] and described briefly below. For ChIP-Seq analysis, the cells were cross-linked with 1% formaldehyde, followed by sonication to fragment chromatin to sizes ranging from 200 to 500 bp. Chromatin fractions from 1 to 5 million cells were used for chromatin immunoprecipitation using 2 micrograms of specific antibodies. Following reverse cross-linking and purification, the ChIP DNA was ligated to Illumina ChIP-Seq adaptors, amplified using the Illumina primers, and sequenced on the Illumina GAII platform. For RNA-Seq assays, total RNA was isolated from cells and reverse transcribed. The cDNA samples were fragmented to 100 to 300 bp by sonication, ligated to Illumina adaptor, and sequenced on the GAII platform, similar to the ChIP-Seq libraries. Sequence reads of 25-bp were obtained, mapped to the human genome (hg18) and processed as described previously [57]. The sequence reads have been deposited in the NCBI Short Read Archive (GSE12646).

## Supporting Information

Figure S1 **Association between USF1 and the hSET1A complex is critical for** *HoxB4* **activation.** (A) K562 cells were transfected with a pREP4-hHoxB4-luc reporter, an expression vector for wild-type USF1 or AUSF1, the dominant negative mutant. A CMV-driven renilla luciferase plasmid was used as a transfection control. Transfected cells were cultured for 48 hrs and lysed for measurement of luciferase activity. (B) K562 cells were transfected with a HDAC1-luc reporter, an expression vector for USF1, and siRNA targeting hSET1A or PRMT1. A CMV-driven renilla luciferase plasmid was used as a transfection control. Transfected cells were cultured for 48 hrs and lysed for measurement of luciferase activity. Data are shown as mean ± SD. ** P<0.01; * P<0.05. (C) K562 cells were transfected with a β-globin-luciferase reporter, an expression vector for USF1, and siRNA targeting PRMT1. A CMV-driven renilla luciferase plasmid was used as a transfection control. Transfected cells were cultured for 48 hrs and lysed for measurement of luciferase activity. Data are shown as mean ± SD. ** P<0.01; * P<0.05. (D) Real-time RT-qPCR analysis of *Hox* gene expression upon hSET1A KD in K562 cells. (E) ChIP analysis of USF1 binding and hSET1A recruitment at the *HoxB4* locus in K562 cells. Data are shown as mean ± SD. *P<0.05; ** P<0.01. (F) ChIP analysis of H3K4me3 levels at the *HoxB4* locus in K562 cells. Data are shown as mean ± SD. * P<0.05. (G) Schematic representation of the GST-USF1 fusion proteins used in GST pull-down assays. (H) ^35^S-labeled ASH2L was incubated with GST and GST-USF1 fusion proteins were pre-absorbed to glutathione-Sepharose beads. (Top) Bound ASH2L was visualized by fluorography. (Bottom) Coomassie blue-stained gel shows relative protein loading. (I) Western blotting analysis of Flag-tagged USF1 and endogenous USF1 in Flag-USF1 overexpressing K562 cells. (J) Reconstitution of the hSET1A complex. SF9 cells were transduced with vectors expressing untagged ASH2L, hSET1A, RBBP5, WDR5, and Flag-tagged HCF1. The baculovirus reconstituted hSET1A complex was purified with Flag antibody and analyzed by immunoblots using antibodies specific to the core components of the hSET1A complex.
(PDF)

Figure S2 **Recruitment of the hSET1A complex, H3K4me3 enrichment, and RNAPII loading correlates with highly active** *β-actin* **expression.** (A) ChIP-seq and RNA-seq analyses of *HoxB4* expression, H3K4 enrichment, and RNAPII loading at the *β-actin* locus in CD34^+^ HSCs. (B) ChIP-seq and RNA-seq analyses of *HoxB4* expression, hSET1A recruitment, RBBP5 binding, H3K4 enrichment, and RNAPII loading at the *β-actin* locus in CD36^+^ hematopoietic progenitors.
(PDF)

Figure S3 **Recruitment the hSET1A complex, H3K4me3 enrichment, and RNAPII loading does not correlate with silenced** *MyoD1* **gene.** (A) ChIP-seq and RNA-seq analyses of *HoxB4* expression, H3K4 enrichment, and RNAPII loading at the *MyoD1* locus in CD34^+^ HSCs. (B) ChIP-seq and RNA-seq analyses of *HoxB4* expression, hSET1A recruitment, RBBP5 binding, H3K4 enrichment, and RNAPII loading at the *MyoD1* locus in CD36^+^ hematopoietic progenitors.
(PDF)

Figure S4 **Molecular characterization of cytokine induced hematopoietic differentiation of ESCs.** (A) ChIP analysis of bivalent H3K4me3 and H3K27me3 marks at HSC-specific and late differentiation stage-specific genes in undifferentiated ES cells. (A) Outlines of the characterization and differentiation of ESCs into hematopoietic stem and progenitor cells. (C) Western blotting assay of the levels of USF1, MLL1, and hSET1A in K562 cells and ESCs. (D) Western blotting assay of the levels of USF1, MLL1, and hSET1A at different stages of induced hematopoietic differentiation. (E) Time course qRT-PCR analyses of the expression levels of early lineage markers upon induced hematopoietic differentiation. (F) Time course qRT-PCR analyses of the expression levels of early hematopoietic transcription factors and primitive hematopoietic marker, *βH1*, upon induced hematopoietic differentiation.
(PDF)

Figure S5 **The recruitment of hSET1A correlates with transcription of the** *HoxB4* **gene during differentiation of ESC.** (A–C)Time course ChIP analyses of USF1 binding (A), hSET1A recruitment (B), and H3K4me3 enrichment (C) at the *HoxB4* locus during different stages of induced ESC hematopoietic differentiation. (D) ChIP assay of MLL1 and MLL2 binding at the *HoxB* locus in K562 cells.
(PDF)

Figure S6 **USF1 regulates ESC pluripotency by controlling mesoderm differentiation.** (A) Real-time RT-qPCR analysis of pluripotency associated *Oct4*, *Nanog*, and *hSET1A* mRNA transcript levels comparing the pcDNA control and the dominant negative AUSF1 overexpressing ES cells. (B) AP staining of the pcDNA control and the dominant negative AUSF1 overexpressing ES cells. (C) Hematopoietic differentiation assay. pcDNA control and AUSF1 overexpressing ES cell clones G5 and F5 were cultured in suspension in the absence of LIF to induce embryonic body (EB) formation for 4 days and then cultured in the presence of SCF to induce hematopoietic differentiation for another 4 days. Shown are EBs from 8 day culture. Scale bar, 100 µm. (D) ChIP assay of USF1 binding and H3K4me3 enrichment at the *Brachyury* (*T*) and *FLK1* promoters in ESCs upon withdrawal of LIF. (E) Real-time RT-qPCR analyses of the expression levels of mesoderm markers in pcDNA control and two AUSF1 overexpressing clones upon withdrawal of LIF. Data are shown as mean ± SD. *P<0.05; ** P<0.01.
(PDF)

Figure S7 **USF1 is required for hematopoietic fate determination and differentiation.** (A) FACS analysis of Sca-1 and c-Kit expressing early hematopoietic stem and progenitor cell population in pcDNA control and AUSF1 overexpressing ESCs upon hematopoietic differentiation at day 13. (B) Percentages of c-kit and Sca-1 double positive HS/PCs 13 days after induced hematopoietic differentiation in the pcDNA transfected control and three AUSF1 expressing mES clones. Data are shown as mean ± SD. ** P<0.01. (C) Flag-tagged USF1 or TAL1 expressing K562 nuclear extracts were incubated with ^35^S-labeled AUSF1 and precipitated with Flag specific antibody. (Top) Bound ^35^S-labeled AUSF1 was visualized by fluorography. (Bottom) Western blotting analysis shows relative Flag-tagged proteins. (D) Gel mobility shift analysis (GMSA) shows that AUF1 does not interfere with the TAL1 DNA binding activity.
(PDF)

Figure S8 **hSET1A regulates hematopoietic differentiation, but not self-renewal of ESCs.** (A) Alkaline phosphatase (AP) staining of the scrambled control and hSET1A KD ES cells. (B) Real-time RT-qPCR analysis of pluripotency associated *Oct4*, *Nanog*, and *USF1* mRNA transcript levels comparing the scrambled control and two individual hSET1A knockdown mES cell clones (Clone C210 and H107). (C) FACS analyses of c-kit and Sca-1 double positive HSCs 13 days after hematopoietic differentiation in the scrambled control and three individual hSET1A knockdown mES cell clones (C210, C208, and H107). (D) Percentages of c-kit and Sca-1 double positive HSCs 13 days after hematopoietic differentiation in the scramble control and three hSET1A knockdown mES clones. Data are shown as mean ± SD. * P<0.05.
(PDF)

Figure S9 **Ectopic expression of USF1 promotes mesoderm differentiation and early hematopoiesis.** (A) Western blotting assay of Flag tagged USF1 protein levels in ESCs harboring vector control or the Flag-tagged USF1 construct. (B) RT-PCR analyses of the expression levels of endoderm and ectoderm markers in pcDNA transfected control and Flag-USF1 overexpressing ES cells upon withdrawal of LIF. Data are shown as mean ± SD. *P<0.05; ** P<0.01. (C) FACS analysis of CD41 and c-Kit expressing early hematopoietic stem and progenitor population in pcDNA transfected control and Flag-tagged USF1 overexpressing ESCs upon hematopoietic differentiation at day 10. (D) Percentages of c-kit and CD41 double positive HS/PCs 10 days after induced hematopoietic differentiation in the pcDNA transfected control and three Flag-USF1 expressing mES clones. Data are shown as mean ± SD. ** P<0.01. (E) Real-time RT-qPCR analysis of primitive hematopoietic markers, ε*y* and β*H1*, upon hematopoietic differentiation comparing control and Flag-USF1 expressing ESCs. Data are shown as mean ± SD. * P<0.05.
(PDF)

Table S1 **Primer sequences for ChIP and RT-qPCR assays.**
(PDF)
