# Supplementary figures and images for: USF1 and hSET1A Mediated Epigenetic Modifications Regulate Lineage Differentiation and HoxB4 Transcription
Source: PLoS Genet. 2013 Jun 6;9(6):e1003524. doi: 10.1371/journal.pgen.1003524 (PMC3675019; doi:10.1371/journal.pgen.1003524)

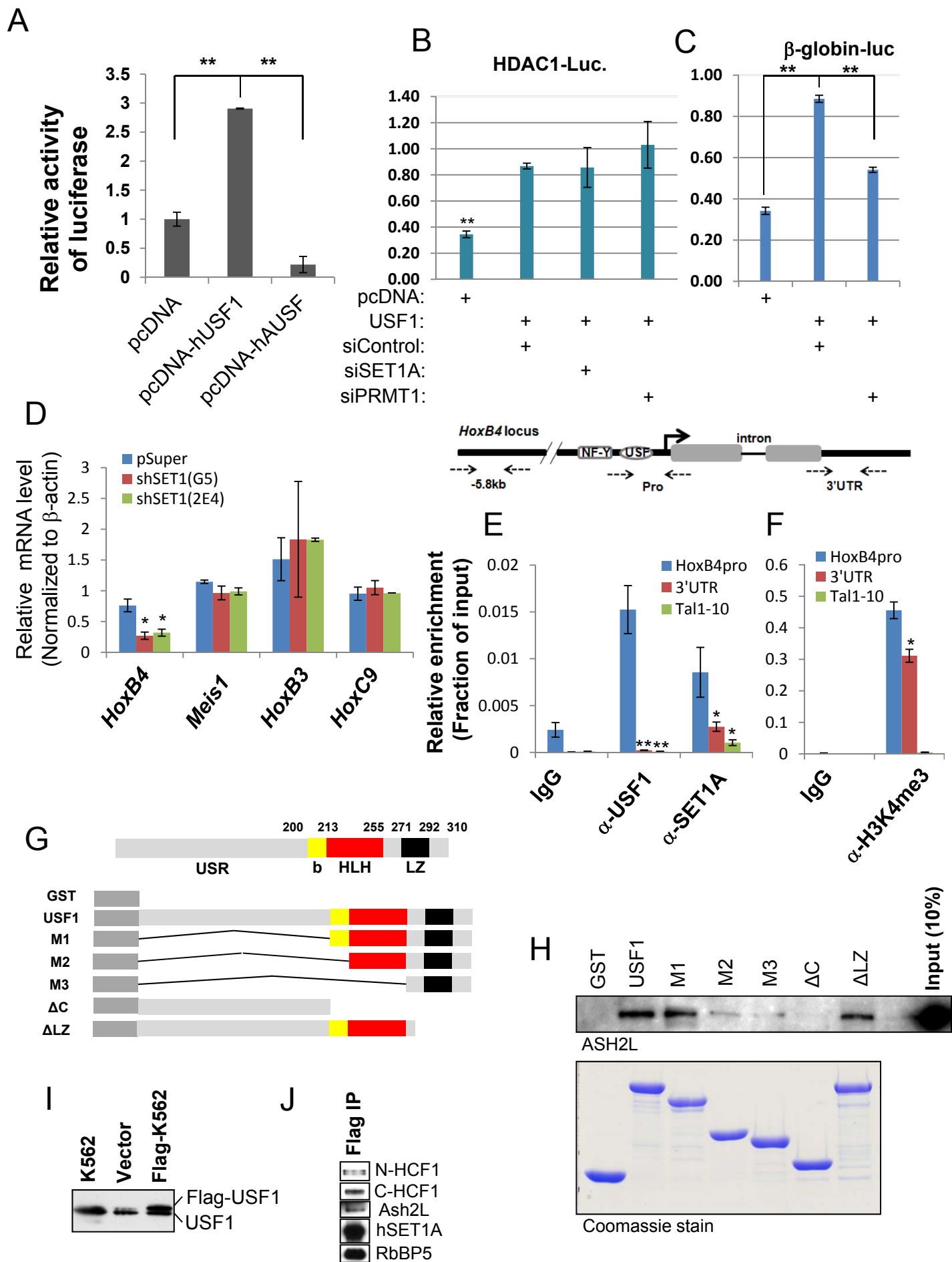

Supplement: Figure S1 — Association between USF1 and the hSET1A complex is critical for HoxB4 activation. (A) K562 cells were transfected with a pREP4-hHoxB4-luc reporter, an expression vector for wild-type USF1 or AUSF1, the dominant negative mutant. A CMV-driven renilla luciferase plasmid was used as a transfection control. Transfected cells were cultured for 48 hrs and lysed for measurement of luciferase activity. (B) K562 cells were transfected with a HDAC1-luc reporter, an expression vector for USF1, and siRNA targeting hSET1A or PRMT1. A CMV-driven renilla luciferase plasmid was used as a transfection control. Transfected cells were cultured for 48 hrs and lysed for measurement of luciferase activity. Data are shown as mean ± SD. ** P<0.01; * P<0.05. (C) K562 cells were transfected with a β-globin-luciferase reporter, an expression vector for USF1, and siRNA targeting PRMT1. A CMV-driven renilla luciferase plasmid was used as a transfection control. Transfected cells were cultured for 48 hrs and lysed for measurement of luciferase activity. Data are shown as mean ± SD. ** P<0.01; * P<0.05. (D) Real-time RT-qPCR analysis of Hox gene expression upon hSET1A KD in K562 cells. (E) ChIP analysis of USF1 binding and hSET1A recruitment at the HoxB4 locus in K562 cells. Data are shown as mean ± SD. *P<0.05; ** P<0.01. (F) ChIP analysis of H3K4me3 levels at the HoxB4 locus in K562 cells. Data are shown as mean ± SD. * P<0.05. (G) Schematic representation of the GST-USF1 fusion proteins used in GST pull-down assays. (H) 35S-labeled ASH2L was incubated with GST and GST-USF1 fusion proteins were pre-absorbed to glutathione-Sepharose beads. (Top) Bound ASH2L was visualized by fluorography. (Bottom) Coomassie blue-stained gel shows relative protein loading. (I) Western blotting analysis of Flag-tagged USF1 and endogenous USF1 in Flag-USF1 overexpressing K562 cells. (J) Reconstitution of the hSET1A complex. SF9 cells were transduced with vectors expressing untagged ASH2L, hSET1A, RBBP5, WDR5, and Fl [file pgen.1003524.s001.pdf]

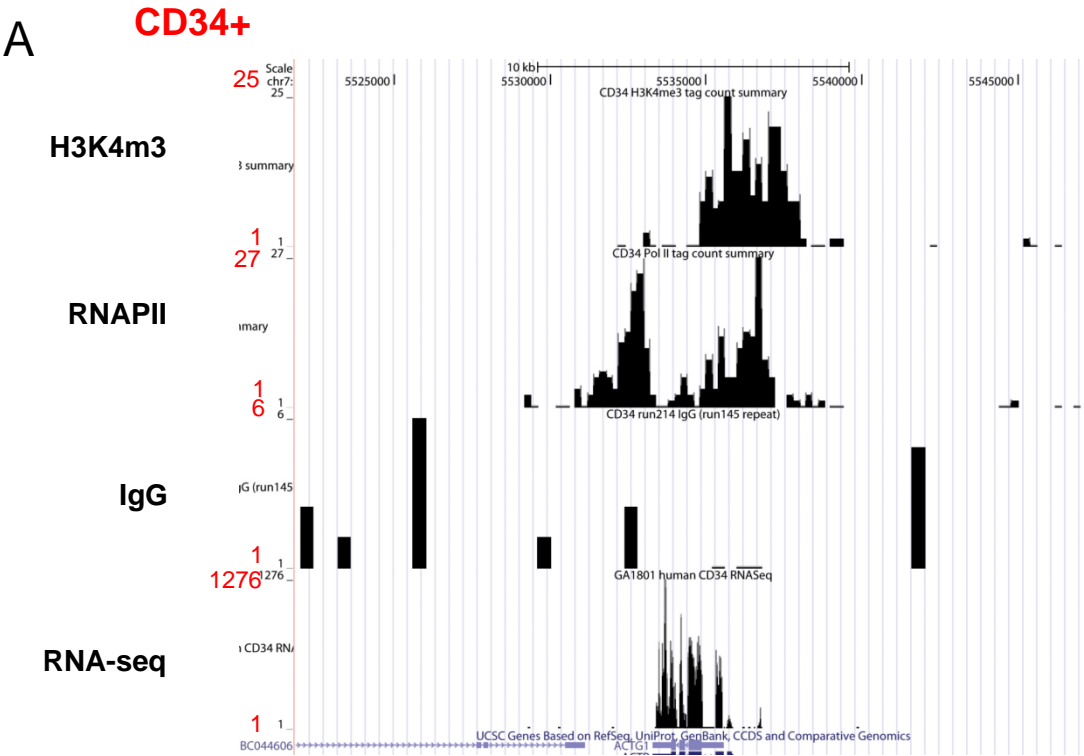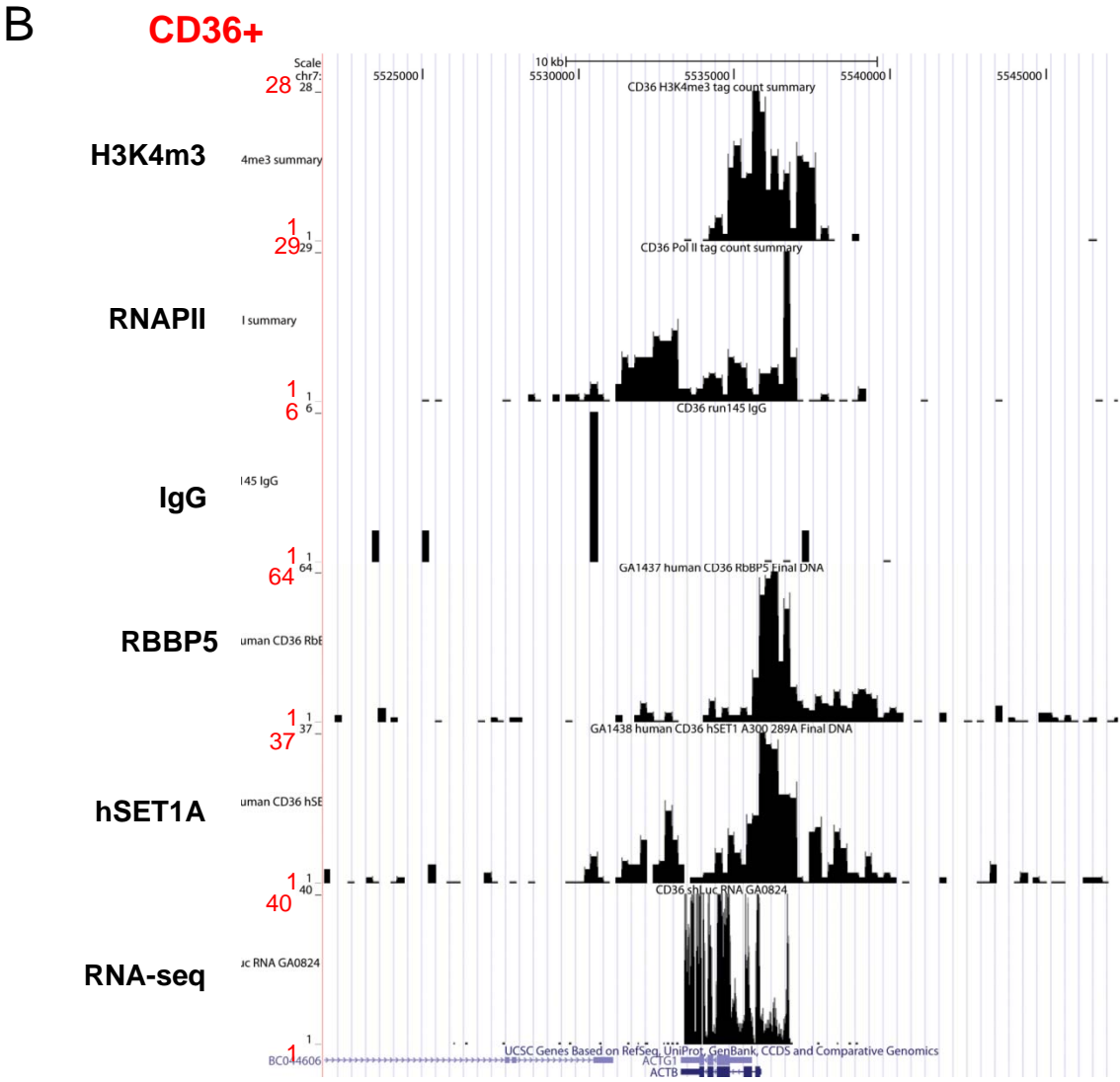

Supplement: Figure S2 — Recruitment of the hSET1A complex, H3K4me3 enrichment, and RNAPII loading correlates with highly active β-actin expression. (A) ChIP-seq and RNA-seq analyses of HoxB4 expression, H3K4 enrichment, and RNAPII loading at the β-actin locus in CD34+ HSCs. (B) ChIP-seq and RNA-seq analyses of HoxB4 expression, hSET1A recruitment, RBBP5 binding, H3K4 enrichment, and RNAPII loading at the β-actin locus in CD36+ hematopoietic progenitors. (PDF) [file pgen.1003524.s002.pdf]

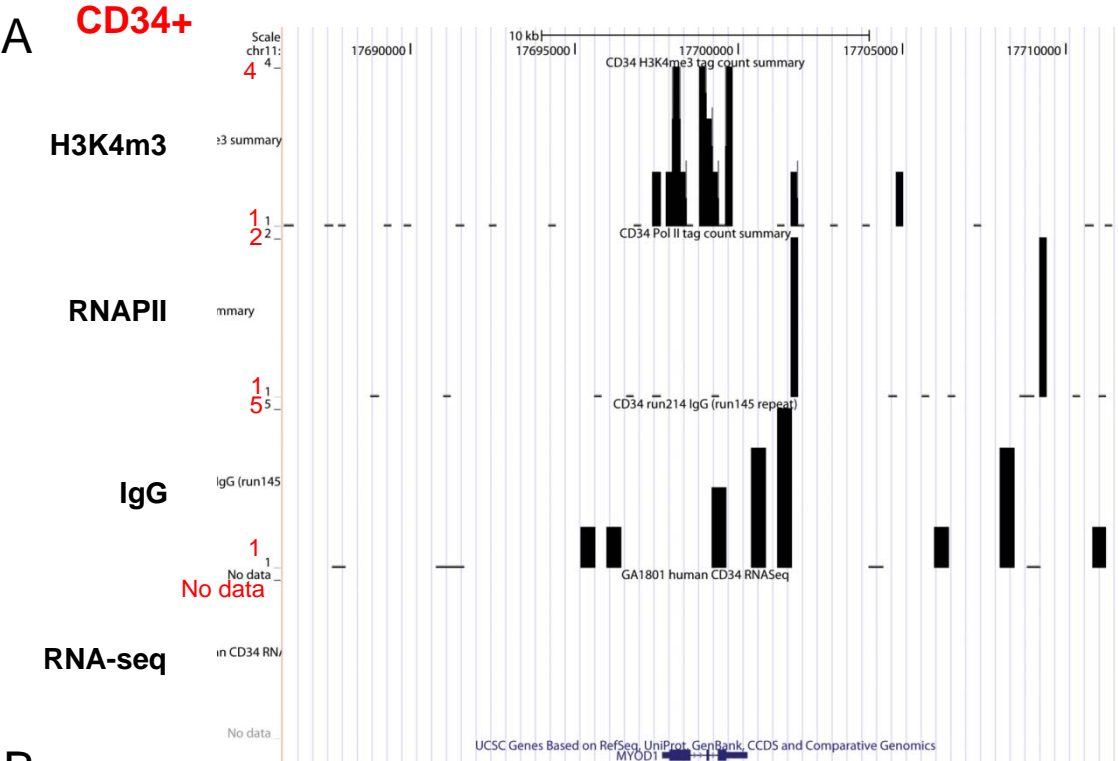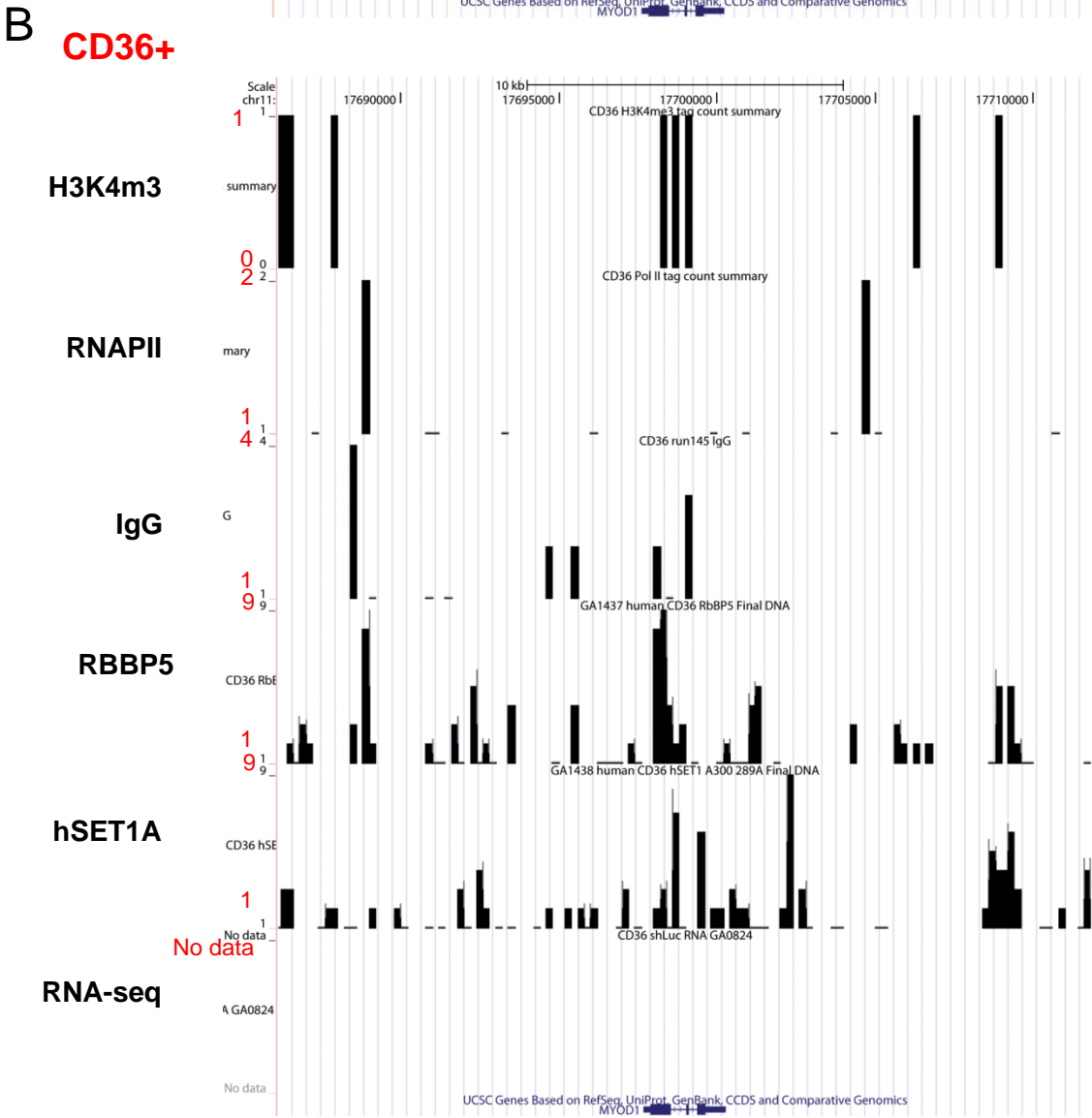

Supplement: Figure S3 — Recruitment the hSET1A complex, H3K4me3 enrichment, and RNAPII loading does not correlate with silenced MyoD1 gene. (A) ChIP-seq and RNA-seq analyses of HoxB4 expression, H3K4 enrichment, and RNAPII loading at the MyoD1 locus in CD34+ HSCs. (B) ChIP-seq and RNA-seq analyses of HoxB4 expression, hSET1A recruitment, RBBP5 binding, H3K4 enrichment, and RNAPII loading at the MyoD1 locus in CD36+ hematopoietic progenitors. (PDF) [file pgen.1003524.s003.pdf]

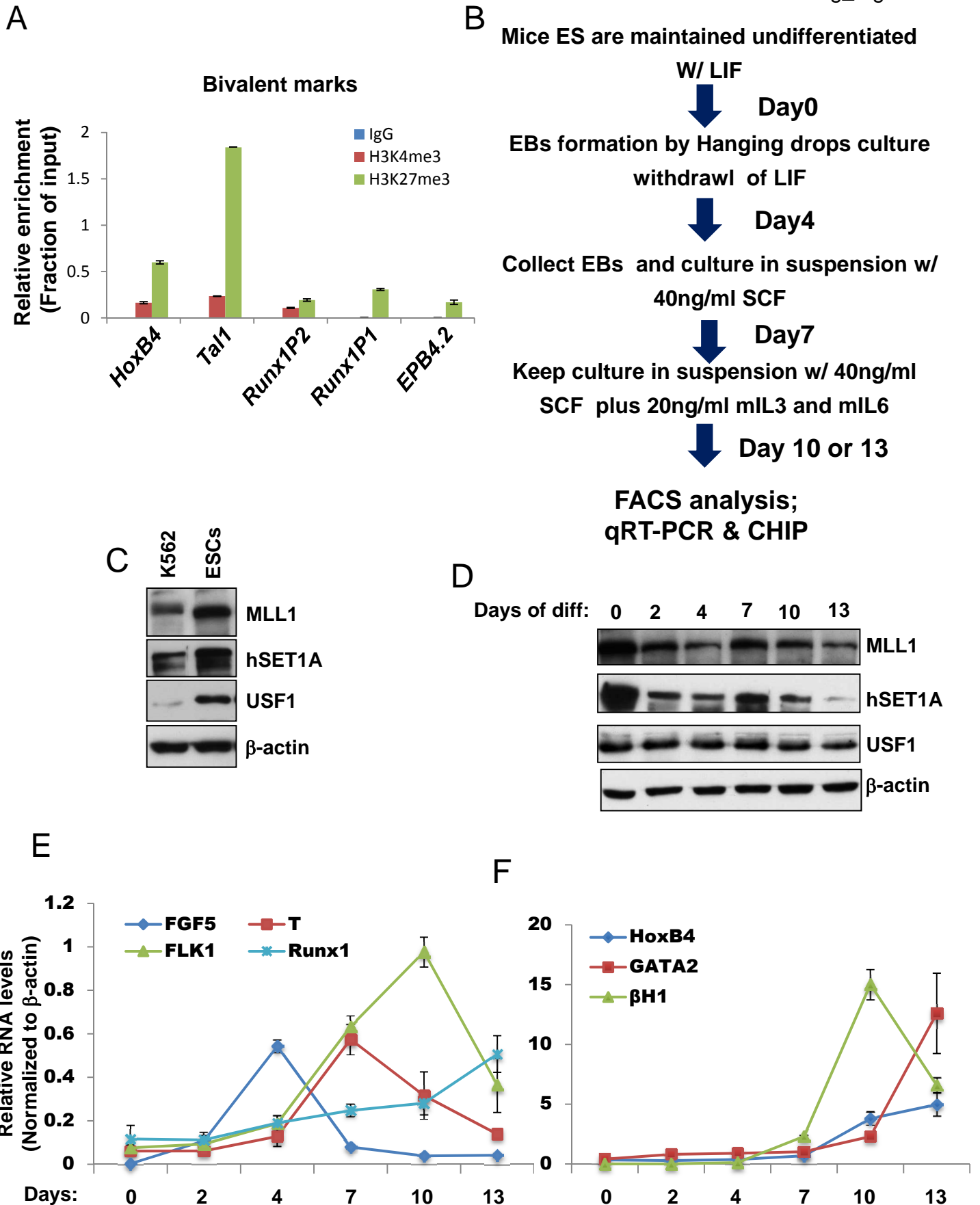

Supplement: Figure S4 — Molecular characterization of cytokine induced hematopoietic differentiation of ESCs. (A) ChIP analysis of bivalent H3K4me3 and H3K27me3 marks at HSC-specific and late differentiation stage-specific genes in undifferentiated ES cells. (A) Outlines of the characterization and differentiation of ESCs into hematopoietic stem and progenitor cells. (C) Western blotting assay of the levels of USF1, MLL1, and hSET1A in K562 cells and ESCs. (D) Western blotting assay of the levels of USF1, MLL1, and hSET1A at different stages of induced hematopoietic differentiation. (E) Time course qRT-PCR analyses of the expression levels of early lineage markers upon induced hematopoietic differentiation. (F) Time course qRT-PCR analyses of the expression levels of early hematopoietic transcription factors and primitive hematopoietic marker, βH1, upon induced hematopoietic differentiation. (PDF) [file pgen.1003524.s004.pdf]

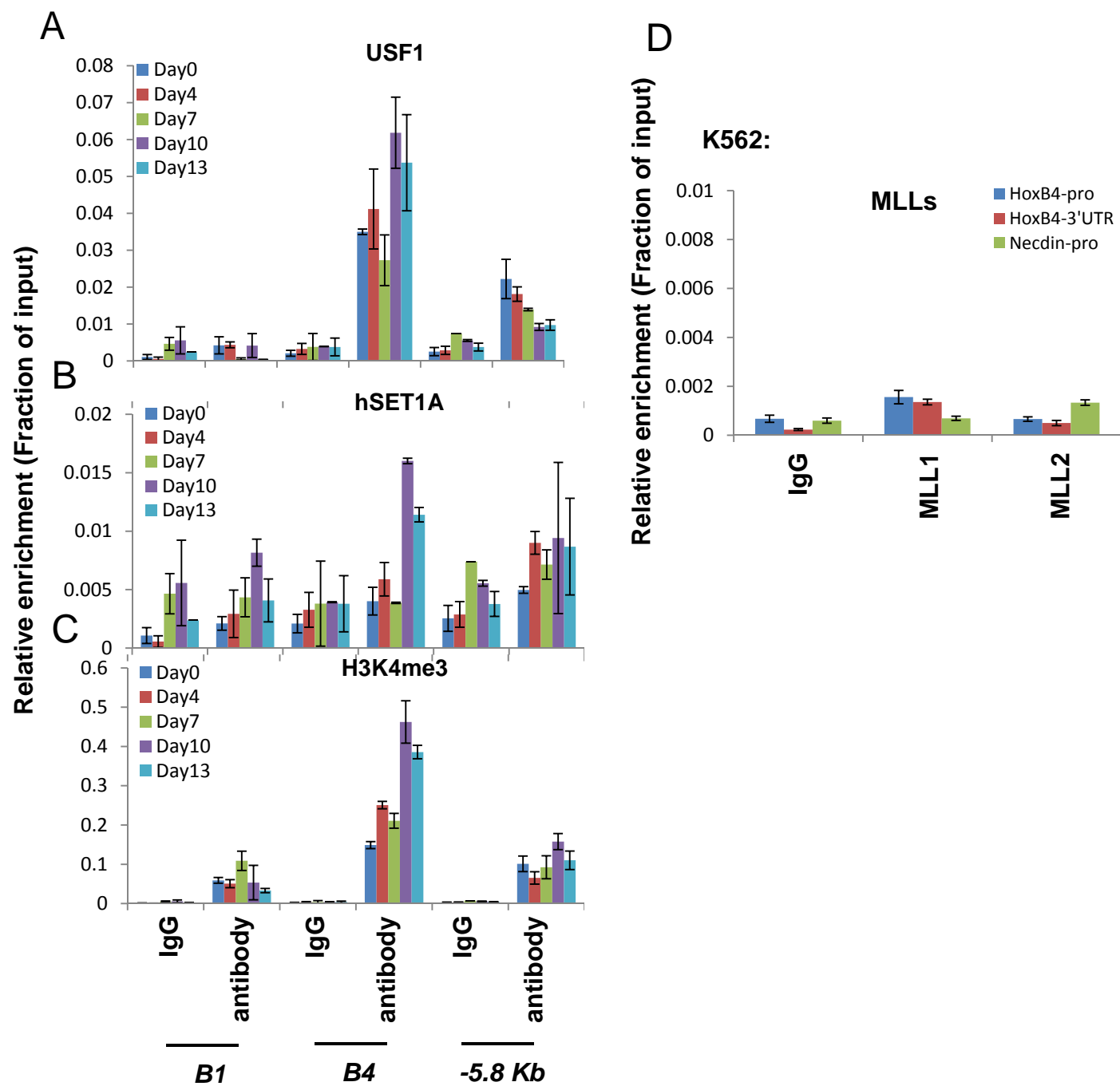

Supplement: Figure S5 — The recruitment of hSET1A correlates with transcription of the HoxB4 gene during differentiation of ESC. (A–C)Time course ChIP analyses of USF1 binding (A), hSET1A recruitment (B), and H3K4me3 enrichment (C) at the HoxB4 locus during different stages of induced ESC hematopoietic differentiation. (D) ChIP assay of MLL1 and MLL2 binding at the HoxB locus in K562 cells. (PDF) [file pgen.1003524.s005.pdf]

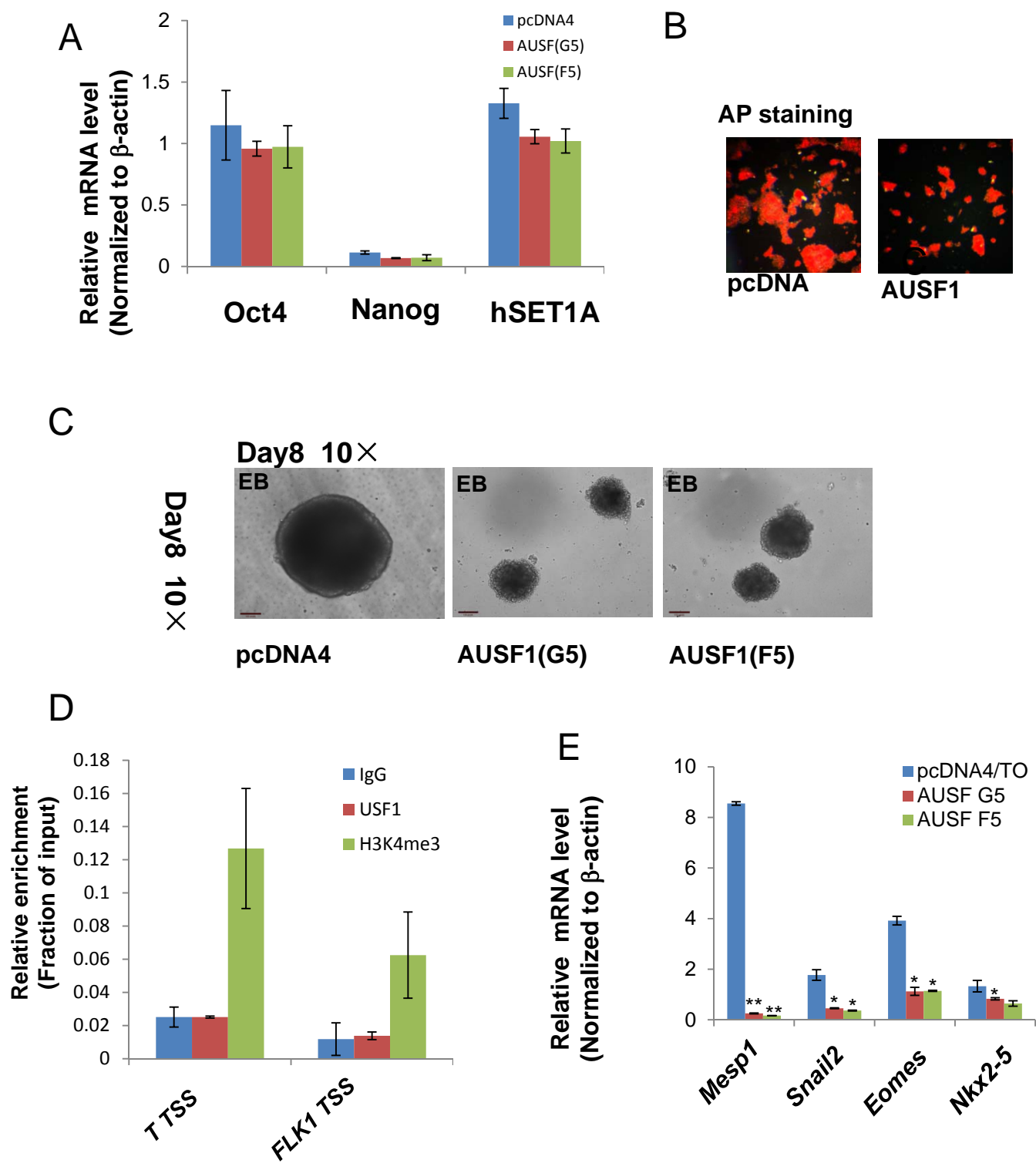

Supplement: Figure S6 — USF1 regulates ESC pluripotency by controlling mesoderm differentiation. (A) Real-time RT-qPCR analysis of pluripotency associated Oct4, Nanog, and hSET1A mRNA transcript levels comparing the pcDNA control and the dominant negative AUSF1 overexpressing ES cells. (B) AP staining of the pcDNA control and the dominant negative AUSF1 overexpressing ES cells. (C) Hematopoietic differentiation assay. pcDNA control and AUSF1 overexpressing ES cell clones G5 and F5 were cultured in suspension in the absence of LIF to induce embryonic body (EB) formation for 4 days and then cultured in the presence of SCF to induce hematopoietic differentiation for another 4 days. Shown are EBs from 8 day culture. Scale bar, 100 µm. (D) ChIP assay of USF1 binding and H3K4me3 enrichment at the Brachyury (T) and FLK1 promoters in ESCs upon withdrawal of LIF. (E) Real-time RT-qPCR analyses of the expression levels of mesoderm markers in pcDNA control and two AUSF1 overexpressing clones upon withdrawal of LIF. Data are shown as mean ± SD. *P<0.05; ** P<0.01. (PDF) [file pgen.1003524.s006.pdf]

**A HSC population:**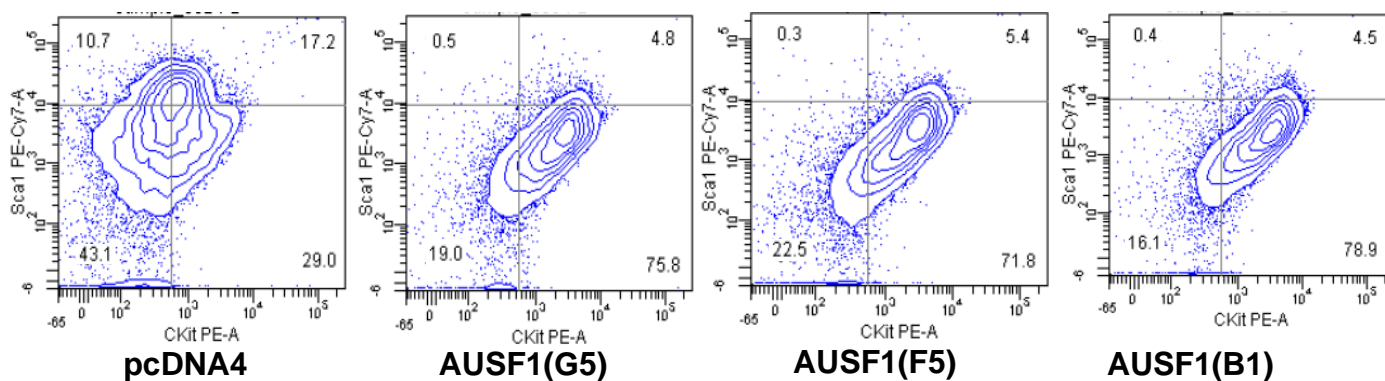**B**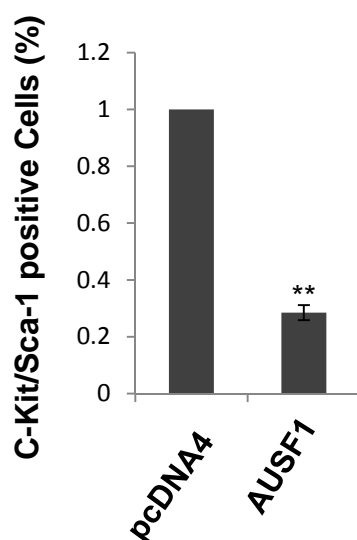**D**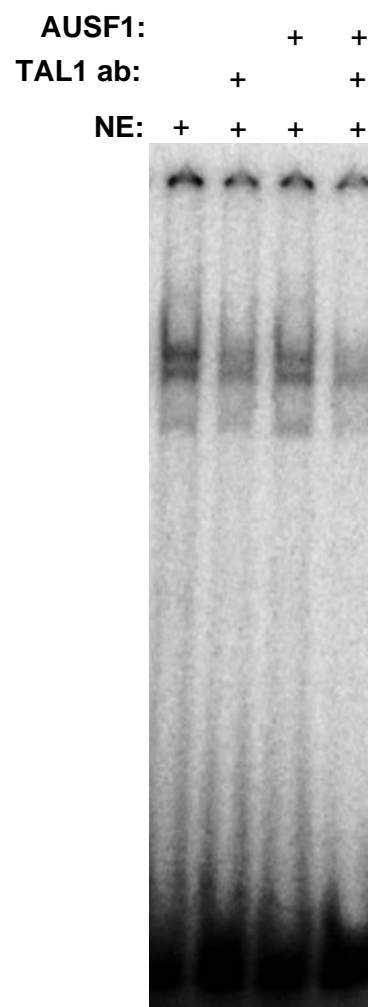**C**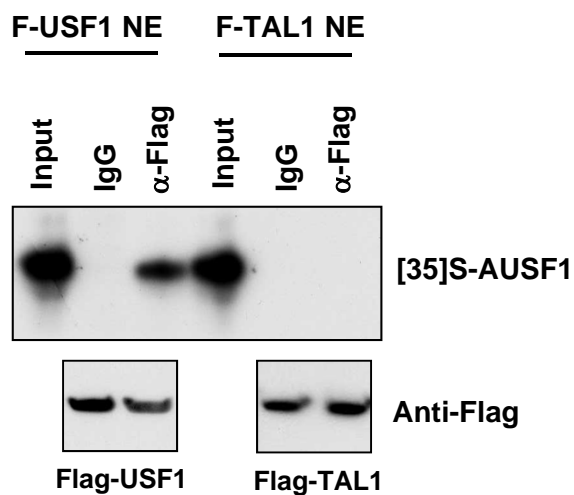

Supplement: Figure S7 — USF1 is required for hematopoietic fate determination and differentiation. (A) FACS analysis of Sca-1 and c-Kit expressing early hematopoietic stem and progenitor cell population in pcDNA control and AUSF1 overexpressing ESCs upon hematopoietic differentiation at day 13. (B) Percentages of c-kit and Sca-1 double positive HS/PCs 13 days after induced hematopoietic differentiation in the pcDNA transfected control and three AUSF1 expressing mES clones. Data are shown as mean ± SD. ** P<0.01. (C) Flag-tagged USF1 or TAL1 expressing K562 nuclear extracts were incubated with 35S-labeled AUSF1 and precipitated with Flag specific antibody. (Top) Bound 35S-labeled AUSF1 was visualized by fluorography. (Bottom) Western blotting analysis shows relative Flag-tagged proteins. (D) Gel mobility shift analysis (GMSA) shows that AUF1 does not interfere with the TAL1 DNA binding activity. (PDF) [file pgen.1003524.s007.pdf]

A

Alkaline Phosphatase (AP) staining

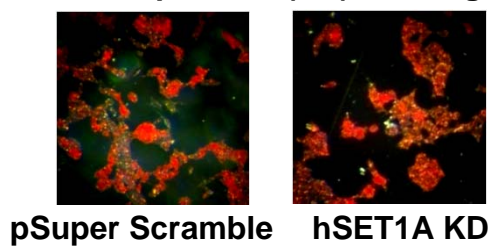

B

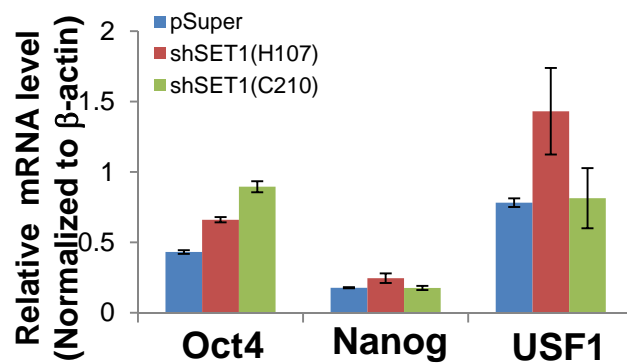

C

HS/PC Population:

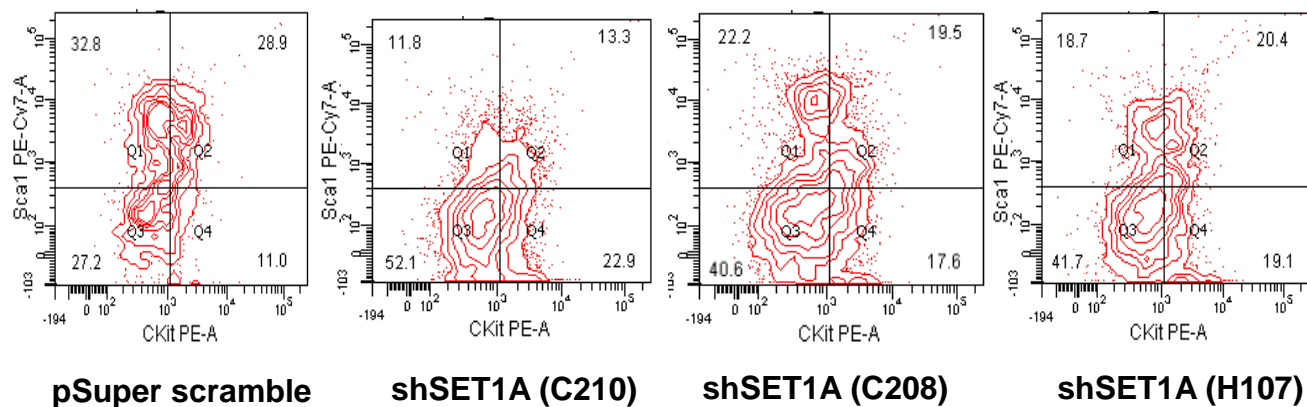

D

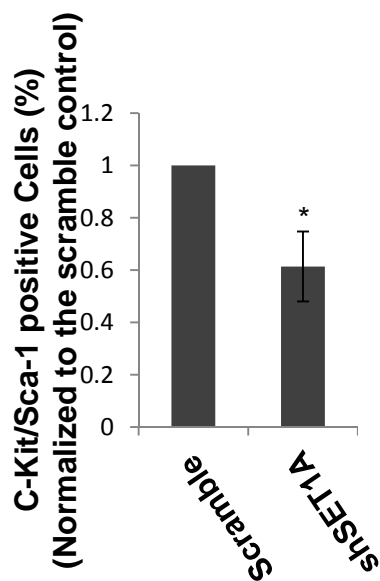

Supplement: Figure S8 — hSET1A regulates hematopoietic differentiation, but not self-renewal of ESCs. (A) Alkaline phosphatase (AP) staining of the scrambled control and hSET1A KD ES cells. (B) Real-time RT-qPCR analysis of pluripotency associated Oct4, Nanog, and USF1 mRNA transcript levels comparing the scrambled control and two individual hSET1A knockdown mES cell clones (Clone C210 and H107). (C) FACS analyses of c-kit and Sca-1 double positive HSCs 13 days after hematopoietic differentiation in the scrambled control and three individual hSET1A knockdown mES cell clones (C210, C208, and H107). (D) Percentages of c-kit and Sca-1 double positive HSCs 13 days after hematopoietic differentiation in the scramble control and three hSET1A knockdown mES clones. Data are shown as mean ± SD. * P<0.05. (PDF) [file pgen.1003524.s008.pdf]

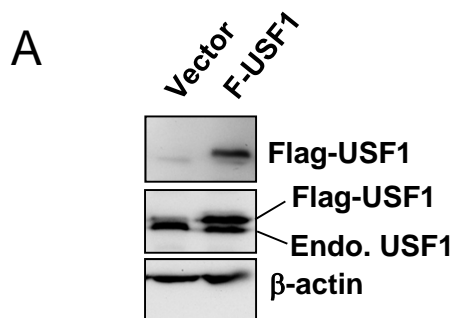

**B**

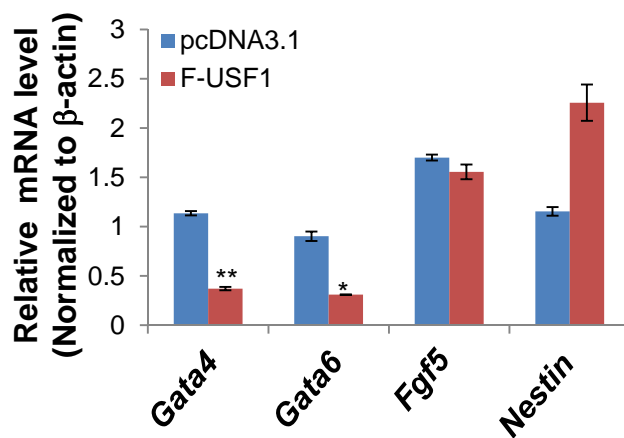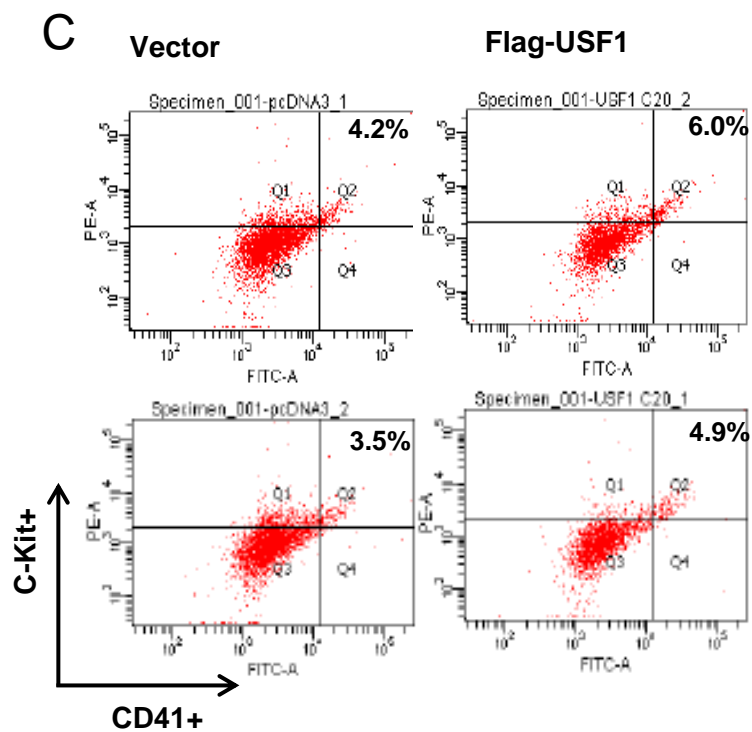

**D**

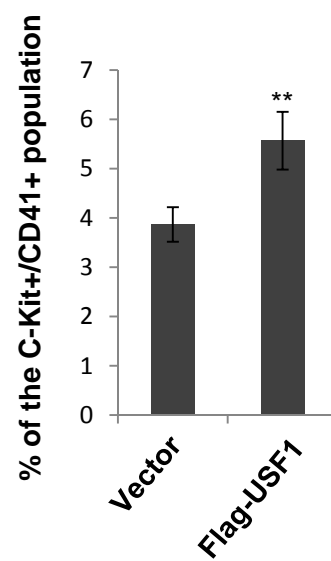

**E**

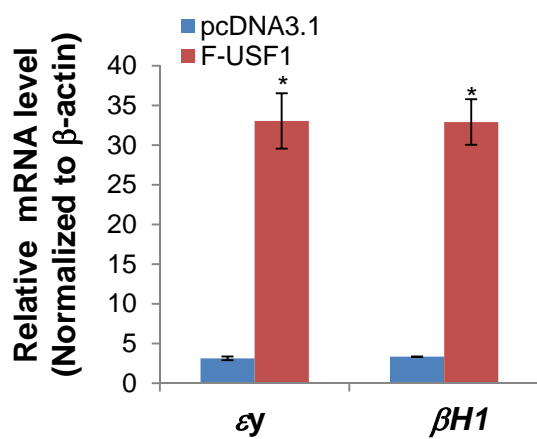

Supplement: Figure S9 — Ectopic expression of USF1 promotes mesoderm differentiation and early hematopoiesis. (A) Western blotting assay of Flag tagged USF1 protein levels in ESCs harboring vector control or the Flag-tagged USF1 construct. (B) RT-PCR analyses of the expression levels of endoderm and ectoderm markers in pcDNA transfected control and Flag-USF1 overexpressing ES cells upon withdrawal of LIF. Data are shown as mean ± SD. *P<0.05; ** P<0.01. (C) FACS analysis of CD41 and c-Kit expressing early hematopoietic stem and progenitor population in pcDNA transfected control and Flag-tagged USF1 overexpressing ESCs upon hematopoietic differentiation at day 10. (D) Percentages of c-kit and CD41 double positive HS/PCs 10 days after induced hematopoietic differentiation in the pcDNA transfected control and three Flag-USF1 expressing mES clones. Data are shown as mean ± SD. ** P<0.01. (E) Real-time RT-qPCR analysis of primitive hematopoietic markers, εy and βH1, upon hematopoietic differentiation comparing control and Flag-USF1 expressing ESCs. Data are shown as mean ± SD. * P<0.05. (PDF) [file pgen.1003524.s009.pdf]
