# Supplementary material for: USF1 and hSET1A Mediated Epigenetic Modifications Regulate Lineage Differentiation and HoxB4 Transcription
Source: PLoS Genet. 2013 Jun 6;9(6):e1003524. doi: 10.1371/journal.pgen.1003524 (PMC3675019; doi:10.1371/journal.pgen.1003524)
Supplement: Table S1 — Primer sequences for ChIP and RT-qPCR assays. (PDF) [file pgen.1003524.s010.pdf]

Table S1. Primer sequences:

	Primer	*Primer sequences*
shRNA	Human SET1A-1	Target: 5’ GGAAAGAGCCATCGGAAAT 3’
	Human SET1A-2	Target: 5’ GACAACAACGAATGAAATA3’
	Human SET1A-3	Target: 5’ CAACGACTCAAAGTATATA 3’
	Mouse SET1A-1	Target: 5’ GCAAGAAGGAGAAAGATAA 3’
	Mouse SET1A-2	Target: 5’ CGGAAAGAGCCATCAGAAA 3’
	shScramble	Target: 5’ TAGCGACTAAACACATCAA 3’
RT-PCR	Mouse β-actin	For: GTGGGCCGCTCTAGGCACCA
		Rev: TGGCCTTAGGGTGCAGGGGG
	Human β-actin	For: AGAAAATCTGGCACCACACC
		Rev: AGAGGCGTACAGGGATAGCA
	HoxB4	For: TGGATGCGCAAAGTTCACG
	(both for human & mouse)	Rev: GGTCTTTTTTCCACTTCATGCG
	Human Meis1	For: TCACACTGGCCTTAAAGAGGA
		Rev: CCGTAATGGGGTAGATCGTC
	Human HoxB3	For: AGTACAAGAAGGACCAGAAGGC
		Rev: TGGAGTGTAAGGCGTTCATG
	Human HoxC9	For: ACGAGGAAGAAGCGCTGCCCC
		Rev: GAGAACCCGGGCCACCTCATA
	Mouse Oct4	For: GGCGTTCTCTTTGGAAAGGTGTTC
		Rev: CTCGAACCACATCCTTCTCT
	Mouse Nanog	For: AGGGTCTGCTACTGAGATGCTCTG
		Rev: CAACCACTGGTTTTTCTGCCACCG
	Mouse USF1	For: GATGAGAAACGGAGGGCTCAACATA
		Rev: TTAGTTGCTGTCATTCTTGATGACG
	Mouse SET1A	For: CAGACGGGCTTGTAGATTCC
		Rev: GAGGCTGTGGTACACTAGG
	Mouse Tal1	For: TAGCCTTAGCCAGCCGCTCG
		Rev: GCGGAGGATCTCATTCTTGC
	Mouse CD41	For: GAACCTGGAAGAAGCTGGCG
		Rev: CTCCACTGTGGCTTCAGCTTG
	Mouse T	For: GCTCTAAGGAACCACCGGTCATC
		Rev: ATGGGACTGCAGCATGGACAG
	Mouse FLK1	For: ACTGCAGTGATTGCCATGTTCT
		Rev: TCATTGGCCCGCTTAACG
	Mouse GATA4	For: GATGGGACGGGACACTACCTG
		Rev: ACCTGCTGGCGTCTTAGATTT
	Mouse GATA6	For: GACGGCACCGGTCATTACC
		Rev: ACAGTTGGCACAGGACAGTCC
	Mouse FGF5	For: ACCCGGATGGCAAAGTCAA
		Rev: CAATCCCCTGAGACACAGCAA
	Mouse Nestin	For: CTGCAGGCCACTGAAAAGTT
		Rev: GACCCTGCTTCTCCTGCTC

	Mouse CD34	For: CTCTAGATCACAGTTCTGTGTCAGC<br>Rev: TAGCACAGAACTTCCCAGCAAAC
	Mouse c-Myb	For: AACGAGCTGAAGGGACAGCA<br>Rev: TGGCATGGTGTTCTCCCAAA
	Mouse CD150	For: ACAGGCGTGCTTATGAAGTAGATG<br>Rev: CCACGGGATCTCTG CTTCATAT
	Mouse εy	For: AACCCTCATCAATGGCCTGTGG<br>Rev: TCAGTGGTACTTGTGGGACAGC
	Mouse βH1	For: AGTCCCCATGGAGTCAAAGA<br>Rev: CTCAAGGAGACCTTTGCTCA
	Mouse MixL1	For: AACCGACGGGCCAAGTC<br>Rev: TCCCCGCCTTGAGGATAAG
	Mouse Mesp1	For: GTTCCTGTACGCAGAAACAGCATC<br>Rev: CAAGGAGGGTTGGAATGGTACAGT
	Mouse Snai2	For: CTTTACCCAGTGGCCTTTCTC<br>Rev: CCACAGATCTTGCAGACACAA
	Mouse Eomes	For: ACCAATAACAAAGGTGCAAACAAC<br>Rev: TGGTATTTGTGCAGAGACTGCAA
	Mouse NKX2-5	For: CAGTGGAGCTGGACAAAGCC<br>Rev: TAGCGACGGTTCTGGAACCA
CHIP	HoxB4 Promoter (both for human & mouse)	For: GCCTCTAACTTTGTTCACTTGAC<br>Rev: AGCCATTAATTTCTGGGAATTGC
	Human HoxB4 3'UTR	For: CCCCGGAAAAATCTATCTGC<br>Rev: CCAAAGCTGAAAACGAGGAG
	mouse HoxB4 -5.8kb	For: AGGGGGCTGTATGTGTGAAG<br>Rev: GGGAGAGAAAATCGTGTGGA
	mouse HoxB1 US	For: AGGAAGATGAATCAAGAAG<br>Rev: CAGAAGCACTAAGAGAAG
	mouse Tal1 Pro1a	For: AGGCTAGACAACGGCCAAAG<br>Rev: AAACAACCCGACTCGGATCTC
	mouse Runx1 P1	For: CACAAATAACAACAGGATCTGAAAGC<br>Rev: TCCCCTGCCTCAGTCTTTTCT
	mouse Runx1 P2	For: CCCAGGCTGATACCGGAAA<br>Rev: TCCCTCAGCTCGTTTGCATA
	mouse EPB4.2	For: GCAGGTCATCTCCAAAGAGC<br>Rev: CGAACCCAACTCTGAACCTC
	Human necdin pro	For: TTCGTCCAAATGAATTACCTGAAG<br>Rev: GGAGCCCCAAAAGAACTCGTA
	mouse Tal1 -10kb	For: CAAATCAGAAGAAAAGACCTGCAA<br>Rev: TTTCATTTTCCTTCCCTCAATCTC
